# Modeling gene expression cascades during cell state transitions

**DOI:** 10.1016/j.isci.2024.109386

**Published:** 2024-03-04

**Authors:** Daniel Rosebrock, Martin Vingron, Peter F. Arndt

**Affiliations:** 1 Department of Computational Molecular Biology, Max Planck Institute for Molecular Genetics, 14195 Berlin, Germany

**Keywords:** Biological constraints, Cell biology, Classification of bioinformatical subject, Systems biology, Transcriptomics

## Abstract

During cellular processes such as differentiation or response to external stimuli, cells exhibit dynamic changes in their gene expression profiles. Single-cell RNA sequencing (scRNA-seq) can be used to investigate these dynamic changes. To this end, cells are typically ordered along a pseudotemporal trajectory which recapitulates the progression of cells as they transition from one cell state to another. We infer transcriptional dynamics by modeling the gene expression profiles in pseudotemporally ordered cells using a Bayesian inference approach. This enables ordering genes along transcriptional cascades, estimating differences in the timing of gene expression dynamics, and deducing regulatory gene interactions. Here, we apply this approach to scRNA-seq datasets derived from mouse embryonic forebrain and pancreas samples. This analysis demonstrates the utility of the method to derive the ordering of gene dynamics and regulatory relationships critical for proper cellular differentiation and maturation across a variety of developmental contexts.

## Highlights

• Fitting pseudotime-ordered expression profiles to interpretable functional forms
• Derivation of transcriptional cascades to define a pseudotime trajectory
• Inference of directionality of regulatory interactions

## Graphical abstract

**Figure:**
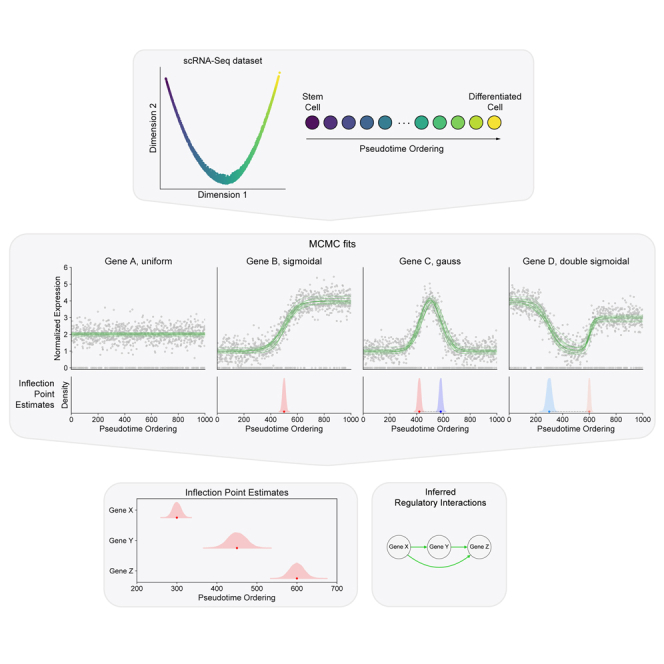

## Introduction

Changes in gene expression underlie the intrinsic molecular processes governing differentiation, enabling cells to change their morphology and function. These changes can occur in part due to extrinsic cues from signaling molecules^1^ or temperature and oxygen levels in the organism’s environment,^2^^,^^3^ as well as intrinsic mechanisms such as the asymmetric distribution of cellular components during cell division.^4^ These processes result in modifying the expression levels of genes that are critical for cell fate specification, most importantly transcription factors, which can initiate or block the expression of downstream target genes, including other transcription factors. The sequential activation and repression of transcription factors and their target genes can give rise to a cascade of gene expression, whereby an initiating event can regulate a hierarchy of downstream genes essential for the cell to acquire subsequent cell states. For example, the *Pax6* → *Eomes* → *Tbr1* transcription factor cascade directs the progression of radial glia to intermediate progenitor to postmitotic projection neuron in the developing cortex,^5^^,^^6^ and the transcription factor cascade initiated by *Neurog3* controls the differentiation of endocrine progenitor cells to mature pancreatic cells.^7^^,^^8^ It is therefore critical to accurately deduce gene expression cascades in order to determine which genes are responsible for specific cell fate changes during differentiation and maturation.

Single-cell RNA sequencing (scRNA-seq) enables sampling the gene expression profile of thousands of cells in an individual sample. However, it is necessary to destroy the cell in order to measure its transcriptome, thereby making it impossible to observe how the cell and its gene expression profile would have altered in the future. Nonetheless, it is possible to order cells along a trajectory which accurately recapitulates the progression of cells as they transition from one cell state to another. This ordering of cells along a trajectory is known as pseudotime, which is essentially a mapping of single-cell transcriptomes to a developmental timeline. Pseudotime methods work under the assumption that cell state changes occur through transitional states, and that these can be measured as gradual shifts in gene expression in individual cells.^9^^,^^10^^,^^11^^,^^12^^,^^13^^,^^14^

Based on the ordering of cells along a pseudotemporal trajectory, it is possible to measure the dynamics of gene expression as cells undergo cell state transitions. Current algorithms typically model gene expression dynamics along pseudotemporal trajectories by fitting their expression profiles using generalized linear models,^12^^,^^15^^,^^16^ with the ultimate goal of determining if gene expression significantly varies as a function of pseudotime. Other methods attempt to deduce pseudotime-dependent gene interactions by calculating a similarity measure between the expression levels of the “present” of one gene, and the “past” of another gene using correlation^17^ or mutual information.^18^ However, these methods do not calculate an explicit ordering of expression dynamics along a pseudotime trajectory, and require user-defined cutoffs for determining meaningful interactions.

Here, we present a method to better understand the cascade of gene expression dynamics underlying cell state transitions. We are interested in answering questions such as, if two genes are up-regulated during a cell state transition, is one gene up-regulated before the other, or are they up-regulated simultaneously? Furthermore, is it possible to estimate a certainty in the timing of their expression dynamics? In this paper, we address these questions by explicitly modeling gene expression over a pseudotime trajectory using a set of functions that reflect biological state switches, and that model the dynamic behaviors of gene expression within cells as they differentiate. We formulate the problem using a Bayesian inference framework and use an ensemble sampler Monte Carlo Markov chain (MCMC) approach^19^ to sample from the posterior distributions over the parameter spaces of the various functions, and determine which model best fits the data. This provides an explicit ordering of genes along a pseudotemporal trajectory based on inflection point estimates, enabling the description of expression dynamics in terms of transcriptional cascades, estimating differences in switch times of gene expression, and annotation of potentially causal gene interactions in gene regulatory networks.

We will introduce our modeling framework in general terms in the first section of the results. A more detailed description is provided in the STAR Methods section. We then apply our method in multiple developmental settings, in which we dissect the transcription factor cascades underlying cortical neurogenesis and pancreatic beta cell development across multiple scRNA-seq datasets. We also show how our method can be used to infer potential upstream regulators of a given gene of interest. Finally, we utilize our method to deduce the gene expression cascade of the Notch signaling pathway in the developing cortex in order to highlight the applicability of our method to gene sets beyond transcription factors. These examples demonstrate the ability of our method to accurately model the dynamics of gene expression during cell state transitions, and highlight the biological insights our method enables.

## Results

### Modeling gene expression dynamics along pseudotime trajectories

The goal of the method presented here is to decide if a state switch (up- to down-regulation or down- to up-regulation) occurs along a pseudotemporal trajectory, and at what pseudotime these switches occur, in order to determine the timing and ordering of activation and repression during cell state transitions. In order to do this, we first define a set of functions which can model a wide variety of expression dynamics, and for which state changes are well defined and interpretable, namely at the inflection points of each function. The functions are then fit to the normalized expression levels for each gene across cells ordered by their relative pseudotempoal ordering. The functions used for fitting are defined as follows,

(Equation 1) $$\begin{array}{l} f_{\text{unif}}\left(t;b\right)=b\text{,}\\ f_{\text{gauss}}\left(t;a,b,t_{0},\sigma \right)=ae^{-\frac{{\left(t-t_{0}\right)}^{2}}{\sigma^{2}}}+b\text{,}\\ f_{\text{sig}}\left(t;k,L,t_{0},b_{\min}\right)=\frac{L}{1+e^{-k\left(t-t_{0}\right)}}+b_{\min}\text{,}\\ f_{\text{dsig}}\left(t;k_{1},k_{2},t_{1},t_{2},b_{\min},b_{mid},b_{\max}\right)=b_{\min}+\frac{b_{mid}-b_{\min}}{1+e^{-k_{1}\left(t-t_{1}\right)}}+\frac{b_{\max}-b_{mid}}{1+e^{-k_{2}\left(t-t_{2}\right)}}\text{.} \end{array}$$

Here, $f_{\text{unif}}$ is a uniform function with b>0, which models the absence of dynamics in gene expression along a pseudotime trajectory. $f_{\text{gauss}}$ is a Gaussian function with parameter constraints a>0, b>0, σ>0, and $1\leq t_{0}\leq N$, with N= number of cells in the pseudotime trajectory. $f_{\text{sig}}$ is a sigmoidal function with parameter constraints L>0, b>0, and $1\leq t_{0}\leq N$. Finally, $f_{\text{dsig}}$ is a double sigmoidal function with the formulation described in the study by Baione et al.^20^ and parameter constraints $b_{\min}>0\text{,}$ $b_{mid}>0\text{,}$ $b_{\max}>0\text{,}$ $k_{1}>0\text{,}$ $k_{2}>0\text{,}$ and $1\leq t_{1}<t_{2}\leq N$. The motivation for using these functions is based on observations from biological scenarios during development.^21^ For instance, during differentiation, genes can display a shift from one steady state to another, which can be modeled using a sigmoidal function. They can also exhibit impulse patterns of up-regulation followed by a return to basal levels, which can be modeled using a Gaussian function. Finally, double sigmoidal functions can model impulse patterns with asymmetric increase and decrease rates and different initial and terminal basal levels, as well as stepwise up and stepwise down expression patterns (Figure S1). We formulate the problem of fitting gene expression profiles in cells ordered along a pseudotime trajectory as a Bayesian inference problem, and estimate parameters for each function using an ensemble sampler MCMC approach^19^ (see STAR Methods). Based on the best-fitting function to the gene expression profiles, genes are ordered according to the relative occurrence of inflection point estimates to provide temporal estimates of gene expression cascades, and regulatory interactions between genes are deduced, enabling a detailed characterization of the molecular processes underlying cellular transitions.

### Transcriptional cascades during cortical neuron differentiation

We first applied our method to differentiating forebrain dorsal neural stem cells during mouse development at embryonic stage e13.5. The input to the method consists of a set of cells ordered by pseudotime, t=1,…,N, and the expression levels (counts) of genes within those cells. Cells from the Atlas of the Developing Mouse Brain^22^ were initially subset to non-dividing forebrain dorsal cells consisting of neural stem cells, intermediate progenitors (IPs), and neurons at embryonic stage e13.5. A pseudotime ordering was estimated using diffusion pseudotime^9^ (Figure S2). All dividing cells were excluded for the pseudotime estimation due to their expression of a transcriptional program that is independent of the underlying cell type, potentially confounding pseudotime estimates.

**Figure 1:**
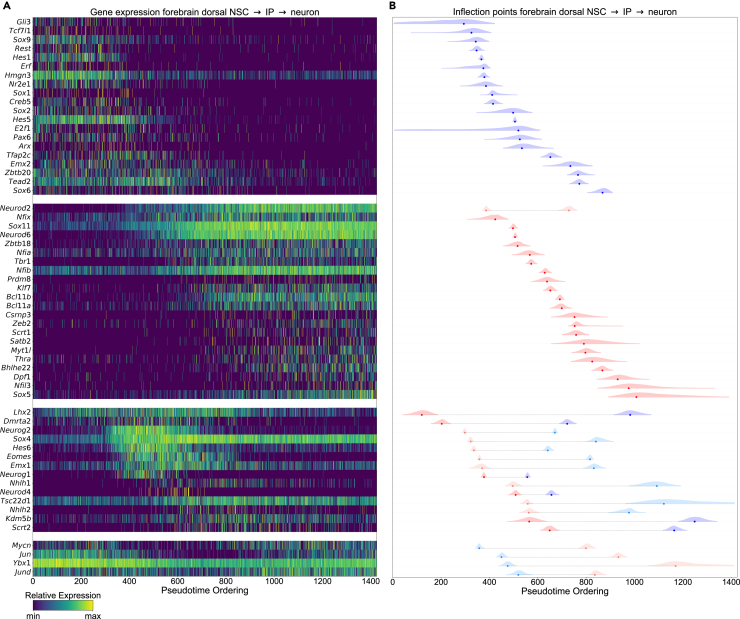
Transcriptional cascades in mouse 13.5 forebrain dorsal cells (A) Gene expression profiles of transcription factors with non-uniform fits are displayed as a heatmap. Genes are grouped according to a state-switch from high to low expression (sigmoidal fit) or stepwise down-regulation (double sigmoidal fit), a state-switch from low to high expression (sigmoidal fit) or stepwise up-regulation (double sigmoidal fit), a transient up (Gaussian or double sigmoidal fit) expression pattern, and transient down (double sigmoidal fit) expression pattern. (B) The inflection point estimates are shown for the same genes as in (A). Inflection point estimates from double sigmoidal fits are shown in light blue and light red, and those from Gaussian and sigmoidal fits in blue and red.

In differentiating cells along the mouse e13.5 forebrain dorsal neural stem cell (NSC) → IP → neuron trajectory, 60 out of 510 (11.8%) transcription factors (derived from the study by Lambert et al.^23^) that were expressed in at least 1% of cells had a non-uniform fit (Figure 1; Table S1). Initially, *Gli3*, a gene that is required for maintaining cortical progenitors in active cell cycle,^24^ was down-regulated in a state-switch manner with a sigmoidal fit, along with *Sox9* and *Hes1*, which are both required for neural stem cell maintenance.^25^^,^^26^ Subsequently, other genes important for neural stem cell maintenance including *Sox1*, *Sox2*, *Hes5*, and *Pax6* were down-regulated. Genes exhibiting a state-switch or stepwise up-regulation included *Neurod2*, *Sox11*, and *Neurod6*, which play a critical role in inducing cell-cycle arrest and neurogenic differentiation in the developing cortex,^27^^,^^28^^,^^29^ followed by *Tbr1* and *Bcl11b*, markers of deep-layer cortical neurons generated during early cortical neurogenesis. Subsequently, *Satb2* and *Bhlhe22*, markers of upper-layer cortical neurons generated during later stages of neurogenesis,^30^ were up-regulated. Interestingly, four transcription factors were found to be transiently down-regulated using a double sigmoidal fit, including *Mycn*, *Jun*, *Ybx1*, and *Jund*. Genes exhibiting a transient up-regulation (Gaussian or double sigmoidal fit) included *Hes6* and *Eomes*, markers of cortical IPs,^31^ as well as *Neurog2* and *Sox4*, which are required for IP cell specification and maintenance via activation of *Eomes*.^32^

These results demonstrate that the functions which best fit the expression profiles of dynamically expressed genes (genes exhibiting a non-uniform fit) largely reflect the known biological role these genes play during differentiation. Furthermore, the relative ordering of inflection point estimates for dynamically expressed transcription factors along the mouse e13.5 forebrain dorsal NSC → IP → neuron trajectory accurately recapitulates known temporal orderings that are essential for the differentiation of cortical neurons. Finally, in order to justify the functional forms we used, we performed a PCA of the gene expression profiles. Genes with a non-uniform fit fill the extremes of the principal component space (Figure S3), indicating that the functional forms we used to model the pseudotime-ordered gene expression profiles are able to capture most of the variability in the data.

### Constructing regulatory interactions during cortical neurogenesis

**Figure 2:**
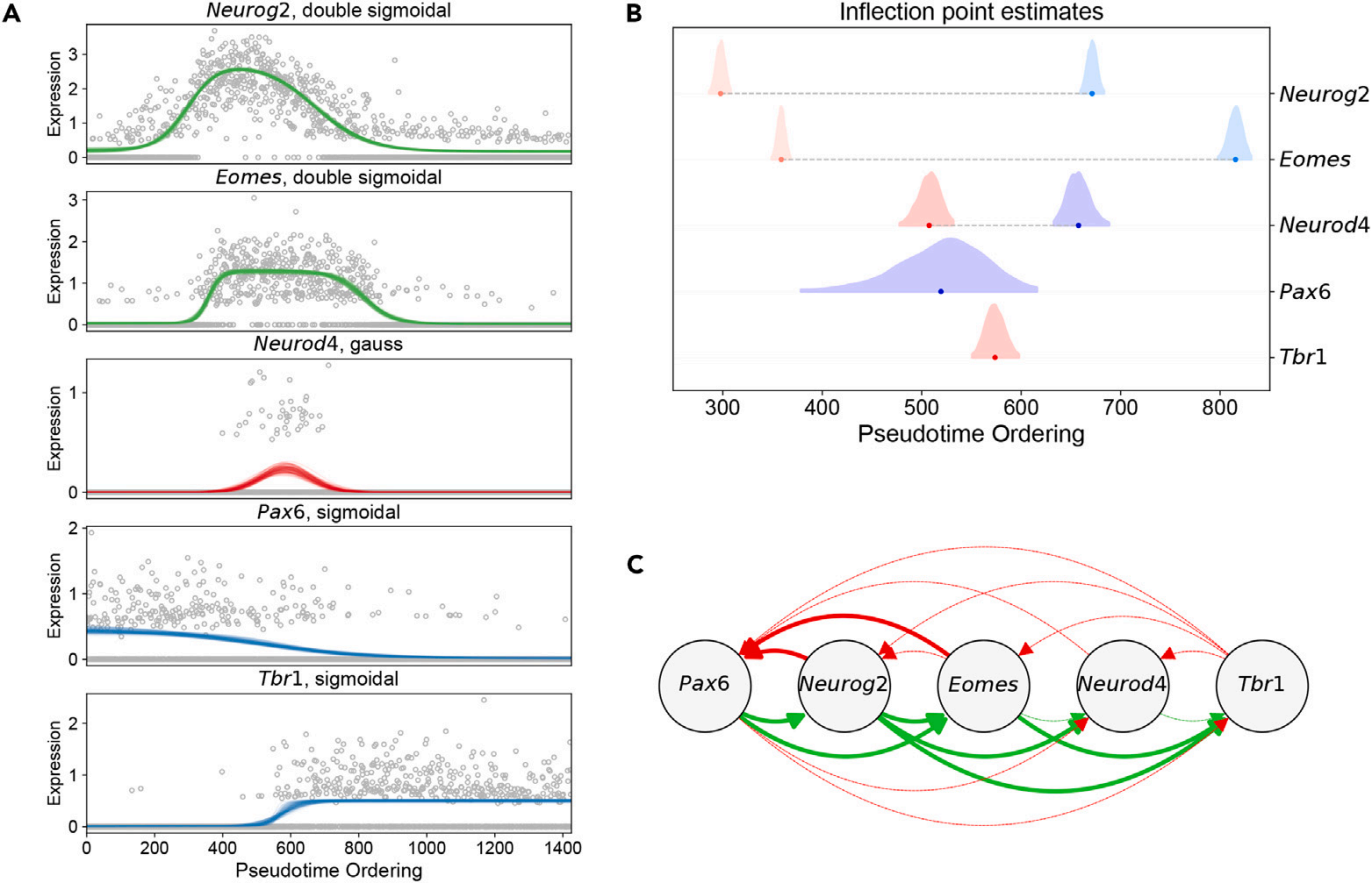
Reconstructing regulatory interactions during mouse e13.5 cortical development (A) Normalized expression levels of essential genes — *Pax6*, *Neurog2*, *Eomes*, and *Tbr1* — forming a regulatory network underlying cortical neuron differentiation, as well as the neural lineage bHLH factor, *Neurod4*, across pseudotime-ordered cells are shown. The curves display a random sampling of the parameters from 100 iterations of the MCMC traces for the best-fitting model for each gene. (B) Inflection point estimates for the genes highlighted in (A). (C) A reconstructed gene regulatory network based on the comparison of inflection points. Positive regulatory interactions which have previously been validated are highlighted as a green solid line, and those which have not been validated as a green dashed line. Similarly, negative regulatory interactions which have previously been validated are highlighted as a red solid line, and those which have not been validated as a red dashed line.

We then compared a set of transcription factors forming an essential regulatory network underlying cortical neuronal differentiation including *Pax6*, *Neurog2*, *Eomes*, and *Tbr1*,^33^ as well as the neural lineage bHLH factor, *Neurod4* (Figure 2A). *Neurog2* and *Eomes* exhibited a transient up-regulation, with both genes having a double sigmoidal fit. *Pax6* and *Tbr1* were fit using a sigmoidal function, with *Pax6* exhibiting a state-switch from high to low expression, and *Tbr1* from low to high expression. *Neurod4* was fit using a Gaussian function, and was specifically expressed transiently in mid-stage *Eomes*^+^ cells.

These genes were then ordered according to the pseudotemporal occurrence of inflection point estimates (Figure 2B), whereby *Neurog2* was found to be up-regulated before *Eomes*, followed by the up-regulation of *Neurod4* and down-regulation of *Pax6*. Subsequently, *Tbr1* was up-regulated, followed by down-regulation of *Neurod4*, *Neurog2*, and finally *Eomes*. *Neurod4* exhibited a brief, transient impulse expression pattern within mid-stage *Eomes*^+^ cells, reflecting previously studied expression patterns of *Neurod4*, which is only expressed in a subset of *Eomes*^+^ cells in the mouse e14.5 cortex.^34^

By comparing inflection point estimates of these genes (see STAR Methods), we were able to reconstruct previously validated regulatory interactions (Figure 2C). The initial up-regulation of *Neurog2* just before *Eomes* up-regulation suggests that *Neurog2* initiates expression of *Eomes* in intermediate progenitors. This relationship has been shown in mouse e13 embryos via electroporation of *Neurog2* cDNA into the ganglionic eminence, where both *Neurog2* and *Eomes* are not expressed, resulting in ectopic expression of *Eomes*.^35^ *Neurog2* has also been shown to directly activate *Neurod4* in cortical IP cells using a luciferase reporter assay,^36^ which we also recapitulate based on the sequential up-regulation of *Neurog2* and *Neurod4*. Furthemore, it has been shown that both *Neurog2* and *Eomes* induce *Tbr1* expression,^36^ which we also infer based on the up-regulation of *Tbr1* following both *Neurog2* and *Eomes*. Interestingly, directly after *Eomes* and *Neurog2* were up-regulated, *Pax6* was down-regulated, suggesting a negative feedback loop, whereby *Pax6* activates both *Eomes* and *Neurog2*, which then both in turn repress *Pax6*, a relationship which has been previously described in the developing mouse cortex.^37^

### Inferring shared upstream regulators of *Eomes*

**Figure 3:**
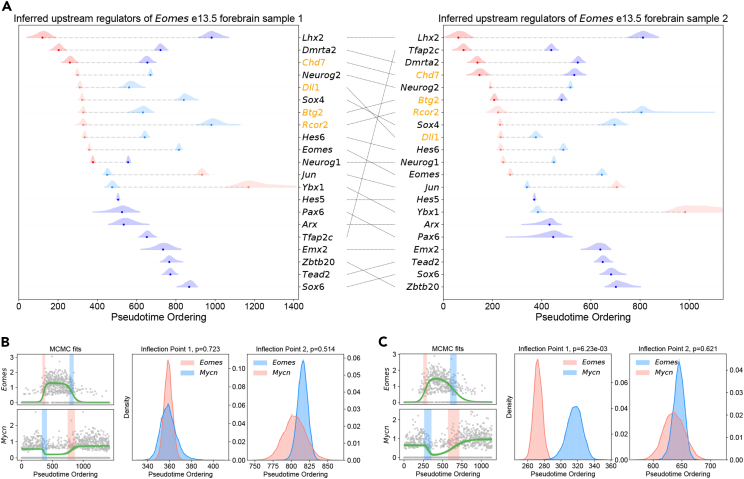
Inferring upstream regulators of *Eomes* across mouse e13.5 embryos (A) The left and right plots show a transcriptional cascade of the shared potential positive regulators of *Eomes* in forebrain dorsal cells of mouse e13.5 embryos across biological replicates. Transcriptional co-activators and co-repressors (derived from the study by Siddappa et al.^38^) are shown in orange, and transcription factors (derived from the study by Lambert et al.^23^) are shown in black. (B) The left panel in the plot displays a random sampling of the parameters from 100 iterations of the MCMC traces for the genes *Eomes* and *Mycn* using the double sigmoidal model, the best-fitting model for both genes. The full range of first and second inflection point estimates for both genes is highlighted as a shaded region, with blue indicating a negative inflection point and red a positive inflection point. The middle and right panels highlight the distribution of first and second inflection point estimates across MCMC iterations, respectively. The right panel highlights the distribution of second inflection point estimates across MCMC iterations. p values were estimated as the percentage of overlapping inflection point estimates across both genes after binning the inflection point estimates across all MCMC iterations to 100 equally spaced bins, starting at the minimum inflection point estimate and ending at the maximum inflection point estimate across both genes. (C) The same plot for (B) in cortical cells of the biological replicate.

We next explored potential upstream regulators of *Eomes* in mouse e13.5 forebrain dorsal cells across two samples in order to deduce high confidence regulators of *Eomes* and determine how robust our method is across biological replicates. We applied our method to forebrain dorsal cells in a mouse e13.5 biological replicate (Figure S4; Table S2). Transcription factors with a positive inflection point occurring simultaneously with or before the first inflection point of *Eomes*, as well as those with a negative inflection point occurring after the first inflection point of *Eomes*, were labeled as positive upstream regulators. We furthermore included all co-activators and co-repressors (derived from the study by Siddappa et al.^38^) that exhibited a transient up-regulation, with the first inflection point occurring simultaneously with or before the first inflection point of *Eomes*. In total, 25 positive upstream regulators were found in the first sample, and 27 were found in the second sample, with an overlap of 21 genes across the two (Figure 3A; Figure S5). Furthermore, the relative ordering of inflection points of these genes along the cortical differentiation trajectory strongly agrees across both datasets, with one exception being *Tfap2c*, which was fit to a sigmoidal function in the first sample, and Gaussian function in the second sample.

Within the set of inferred transcription factors regulating *Eomes* expression were *Neurog2* and *Pax6*, which are known to directly activate *Eomes* in the developing mouse neocortex, as described in the previous section. The co-regulators *Dll1*, a key ligand for activating Notch signaling, and *Chd7*, a chromatin remodeler, have also been implicated in the formation of IP cells,^39^^,^^40^ although their role as a co-activator of *Eomes* has not been established to our knowledge. These results validate the utility of our method in discovering upstream regulators of a given gene of interest. The remaining potential activators of *Eomes* warrant further experimental validation.

Furthermore, the genes that repress *Eomes* in maturing IP cells, thereby enabling the differentiation of these cell types into neurons, are largely unknown.^33^ The transcription factor *Mycn*, a gene critical for normal brain development,^41^ has been shown to down-regulate *Eomes* in neuroblastoma cell lines^42^; however, its role in regulating *Eomes* expression in maturing IP cells is not well understood. In differentiating cells along the forebrain dorsal NSC → IP → neuron trajectory in both mouse e13.5 samples, *Mycn* was expressed in a transient down-regulation pattern and best fit using a double sigmoidal function (Figures 3B and 3C). In both samples, *Mycn* up-regulation occurred simultaneously with *Eomes* down-regulation, signifying that *Mycn* may play a role in the differentiation of cortical neurons by down-regulating *Eomes* in maturing IPs.

### Dissecting Notch signaling during cortical neurogenesis

**Figure 4:**
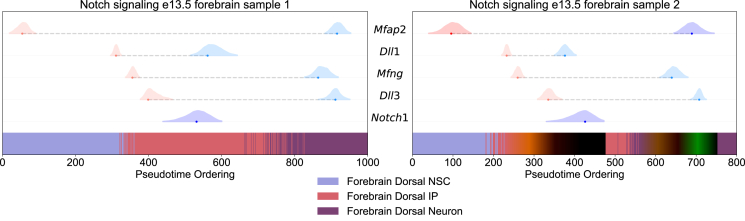
Notch signaling cascade in mouse e13.5 embryos The left and right plots show a transcriptional cascade of the shared ligand-receptor pairs involved in Notch signaling in cells along the forebrain dorsal NSC → IP → neuron trajectories in mouse e13.5 embryos across biological replicates. Annotated cell types are highlighted below.

To demonstrate the applicability of our method to genes beyond transcription factors, we investigated the dynamics of Notch signaling along the forebrain dorsal NSC → IP → neuron trajectory in e13.5 mouse embryos. Shared dynamically expressed genes involving ligand-receptor pairs of Notch receptors from the study by Shao et al.^43^ in both embryonic samples were estimated (Figure 4).

In both samples, *Mfap2*, which can interact with the extracellular domain of *Notch1*,^44^ however whose role is poorly understood in the regulation and differentiation of cortical NSCs, was up-regulated within forebrain dorsal NSCs, and down-regulated in neuronal cells. This indicates that *Mfap2* may play a general role in Notch signaling within differentiating cortical NSCs, whose actions are not specific to a given cell type. *Dll1* was up-regulated in early IPs, followed by the up-regulation of *Dll3* in later stage IPs, confirming the selective basal expression of *Dll3* from *in vivo* studies.^33^ Furthermore, *Mfng*, a glycosyltransferase which increases the ability of *Notch1* to bind to *Dll1*,^45^ was up-regulated shortly after *Dll1* up-regulation in both samples within IPs, indicating that this gene becomes activated sequentially after the activation of *Dll1*. *Dll1* was then down-regulated within IPs, suggesting that this gene is not essential for further IP differentiation into neurons. Finally, *Notch1* was down-regulated in maturing IPs, followed by down-regulation of *Mfap2*, *Mfng*, and *Dll3* in neurons. These results highlight the ability of our method to dissect the complex dynamics of signaling pathways within differentiating cell types.

### Transcriptional cascades in mouse pancreatic beta cell development

**Figure 5:**
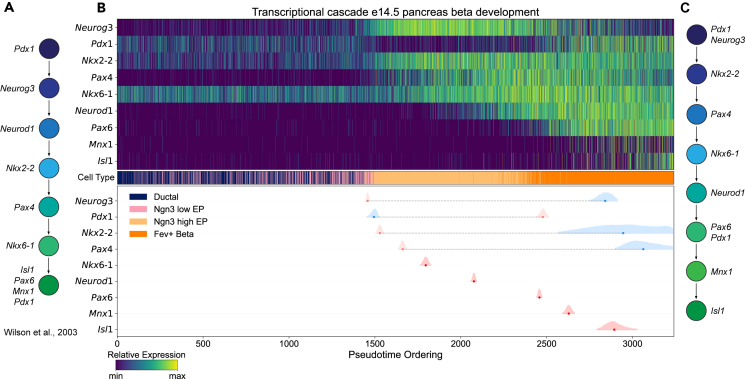
Gene expression cascades in developing mouse e14.5 pancreatic beta cells (A) Schematic diagram of the previously characterized gene expression cascade in developing pancreatic beta cells, based on the study by Wilson et al.^8^ (B) The heatmap in the upper panel highlights the expression profiles of transcription factors ordered by the occurrence of their first inflection points. Inflection point estimates are highlighted in the plot below using the same ordering, with double sigmoidal fits shown in light blue and light red, and those from Gaussian and sigmoidal fits in blue and red. The annotated cell type for each cell in the trajectory is highlighted in the middle. (C) Modified gene expression cascade based on inflection point estimates from (B).

To demonstrate the utility of our method in other developmental contexts, we applied our method to a scRNA-seq dataset of pancreatic cells derived from mouse e14.5 embryos,^46^ subsetting to cells belonging to the beta cell lineage. When measuring the expression dynamics of a set of genes known to play an essential role in the specification and maturation of pancreatic beta cells,^8^ we find a well-defined transcriptional cascade which largely agrees with previously characterized gene expression cascades (Figure 5A). Interestingly, we find one exception to this cascade, *Neurod1*, which is up-regulated at a later stage of beta cell maturation than previously reported (Figures 5B and 5C). We are also able to measure the sequential up-regulation of *Pax6* and *Pdx1*, followed by *Mnx1*, and ending with the insulin gene expression regulator *Isl1*, thereby providing a more explicit ordering of the expression cascade in maturing beta cells than previously established. Furthermore, with this approach, we can model the expression dynamics of all transcription factors (Figure S6; Table S3), enabling a detailed overview of the full gene expression cascade underlying pancreatic beta cell differentiation.

## Discussion

In this paper, we explored an approach to model the gene expression dynamics in cells ordered by a pseudotime trajectory using a fully Bayesian framework. This framework enabled us to fit the gene expression profiles of cells undergoing cell state transitions to a set of functions that are able to model complex transcriptional dynamics. From these fits, we were able to order genes along a gene expression cascade which describes the molecular dynamics underlying cell state transitions, and deduce regulatory interactions.

We first applied the method to differentiating forebrain dorsal neural stem cells into neurons in mouse e13.5 embryos. By ordering transcription factors by the relative occurrence of inflection point estimates, we were able to reconstruct the transcriptional cascades underlying neuronal differentiation within the developing cortex, and model the dynamics of gene expression for all genes along the trajectory. However, genes can undergo further dynamic changes including post-transcriptional and post-translational modifications, and localization changes within the cell, all of which can have a large impact on function and regulation. While transcriptomics data are unable to identify these changes, the dynamics we uncover from gene expression data can still shed light on their regulatory roles.

By comparing the relative timing of expression dynamics of the transcription factors *Pax6*, *Neurog2*, *Eomes*, *Neurod4*, and *Tbr1*, which form a regulatory network underlying cortical neuron differentiation, we were able to infer known causal interactions. However, reconstructing a gene regulatory network using all genes with a non-uniform fit would lead to many false positives, in part due to the simultaneous activation of multiple pathways involving different genes. Thus, we believe one of the main utilities of our approach is to infer the directionality of regulatory interactions, especially in cases where an interaction has been measured but the directionality is unknown.

We then identified potential upstream positive regulators of *Eomes*, an essential gene for the formation of IPs. Subsetting to genes which have similar dynamics across biological replicates revealed a set of high-confidence potential upstream regulators. Not only did we recover validated activators of *Eomes*, such as *Pax6* and *Neurog2*, but we also detected a number of other transcription factors whose roles in *Eomes* activation have not been fully characterized. The enrichment of known DNA-binding motifs of these transcription factors in the promoter and enhancer regions of *Eomes* may provide further evidence for the regulatory role of these genes in *Eomes* expression. We also identified a potential negative regulator of *Eomes*, the transcription factor *Mycn*, whose role in cortical IP maturation has not been fully explored. Wet lab experiments, such as knockin or knockout experiments, or chromatin immunoprecipitation sequencing experiments, would need to be performed in order to validate the roles of these transcription factors in the regulation of *Eomes* expression.

We further demonstrated the applicability of our method to genes beyond transcription factors by comparing the expression dynamics of genes involved in the Notch signaling pathway. This analysis revealed a sequential up-regulation of the Notch receptor ligand *Dll1* in early IPs, followed by *Mfng*, and finally *Dll3* in maturing IPs. This activation cascade supported the selective expression of *Dll1* and *Dll3* in apical and basal IPs, respectively, further demonstrating the utility of comparing genes according to inflection point estimates to dissect signaling pathways.

We also applied our method to differentiating pancreatic beta cells in mouse e14.5 embryos. Based on this analysis, we were able to reconstruct a gene expression cascade that defines beta cell maturation. In this analysis, we highlighted a gene that deviated from the established literature, *Neurod1*, whose up-regulation along the cascade occurred later during beta cell development than previously established. Follow-up experiments are needed to validate these findings.

In order to place our method in a broader context, we compared our results with Monocle 3^12^ and tradeSeq,^16^ which perform statistical tests to determine if a gene is differentially expressed along a pseudotime trajectory, in cells from the e13.5 forebrain dorsal NSC → IP → neuron trajectory. While the overwhelming majority of genes with a non-uniform fit from our method were also found to be significantly differentially expressed by these two methods, both methods detected at least six times more genes to be significant compared to our method (Figure S7). Thus, we conclude that our method is more stringent in detecting genes exhibiting dynamic changes along a trajectory. Furthermore, while the relative ordering of gene expression dynamics along a trajectory is not readily available using these two methods, we are able to explicitly infer this using our method based on inflection point estimates. Similar to our method, the authors of the original diffusion pseudotime publication used derivative estimates of smoothed gene expression profiles to order gene dynamics along a pseudotime trajectory.^9^ However, the authors only used derivative estimates to measure switch-like transitions and not transient up or down transitions, and only provide point estimates of these transitions. We are able to model a higher variety of transitions, and based on the MCMC samplings, quantify the uncertainty in the timing of these transitions using the posterior distribution of the parameter fits.

To measure the dependence of our method on the pseudotime method used to order cells, we ran our method on the pseudotime-ordered cells from the e13.5 forebrain dorsal NSC → IP → neuron trajectory using both Slingshot^10^ and Monocle 3,^12^ and compared them with the diffusion pseudotime estimates (Figure S8). Overall, the fits were largely consistent independent of the pseudotime method used to order the cells, indicating that our method is robust to fluctuations in pseudotime estimates and underlying pseudotime method.

While we focused specifically on cells along the forebrain dorsal NSC → IP → neuron trajectory, and pancreatic beta cell development, the method presented in this paper can be applied to any scRNA-seq dataset where cells can be ordered along a pseudotime trajectory. Our method is able to reconstruct transcriptional cascades in order to deduce critical genes for cell state transitions. It is also able to predict regulatory interactions, as well as gene interactions involved in different signaling pathways. Therefore, we believe this approach can provide useful insights into the molecular underpinnings involved in a variety of developmental biology contexts.

### Limitations of the study

We do not perform any experiments to validate the derived regulatory interactions from differentiating mouse e13.5 forebrain dorsal neurons. Furthermore, deriving regulatory interactions based on all genes with a non-uniform fit along a trajectory would lead to many false positive interactions. Therefore, incorporating other databases and/or scATAC-seq datasets to measure the enrichment of DNA-binding motifs of a transcription factor in the promoter or enhancer regions of an inferred target would provide more evidence of the interaction, which we plan to incorporate in future research.

## STAR★Methods

### Key resources table

**Table:** 

REAGENT or RESOURCE	SOURCE	IDENTIFIER
**Deposited data**		

Atlas of the Developing Mouse Brain	La Manno et al.^22^	http://mousebrain.org/development/downloads.html
Mouse Pancreas Endocrinogenesis Dataset	GSE132188^46^	http://www.ncbi.nlm.nih.gov/geo/query/acc.cgi?acc=GSE132188

**Software and algorithms**		

python	www.python.org	https://www.python.org/downloads/release/python-397/
scanpy	pypi.org	https://pypi.org/project/scanpy/1.9.1/
matplotlib	pypi.org	https://pypi.org/project/matplotlib/3.5.1/
numpy	pypi.org	https://pypi.org/project/numpy/1.21.6/
scipy	pypi.org	https://pypi.org/project/scipy/1.8.0/
emcee	emcee.readthedocs.io	https://emcee.readthedocs.io/en/stable/user/install/
R	cran.r-project.org	https://cran.r-project.org/src/base/R-4/R-4.0.2.tar.gz
Monocle 3	bioconductor.org	https://cole-trapnell-lab.github.io/monocle3/docs/installation/
Slingshot	bioconductor.org	https://bioconductor.org/packages/devel/bioc/vignettes/slingshot/inst/doc/vignette.html
tradeSeq	bioconductor.org	https://bioconductor.org/packages/devel/bioc/vignettes/tradeSeq/inst/doc/tradeSeq.html

### Resource availability

#### Lead contact

Further information and requests should be directed to and will be fulfilled by the lead contact, Daniel Rosebrock (rosebroc@molgen.mpg.de).

#### Materials availability

This study did not generate new unique reagents.

#### Data and code availability

• This paper analyzes existing, publicly available data. These accession numbers for the datasets are listed in the key resources table.
• All original code has been deposited at https://github.com/daniel-rosebrock/transcriptional_cascades and is publicly available as of the date of publication.
• Any additional information required to reanalyze the data reported in this paper is available from the lead contact upon request.

### Method details

#### Processing scRNA-Seq of mouse e13.5 forebrain dorsal samples

The raw count data from the Atlas of the Developing Mouse Brain^22^ was downloaded from http://mousebrain.org/development/downloads.html. The raw count data was loaded into scanpy^47^ for downstream analyses. Cells were then initially subset to samples corresponding to e13.5 embryos derived from the forebrain dorsal tissue (labeled as ‘ForebrainDorsal’ in the metadata), and further subset to ‘Radial glia’ and ‘Neuron’ cell types. The first sample (‘SampleName’ = ‘G23’) and second sample (‘SampleName’ = ‘G9’) were analyzed separately. Initially, in both sample, cells with a DoubletFinderPCA^48^ score above 0.5 were filtered to remove potential doublets. Following this, the count data was normalized using scanpy’s ‘normalize_total’ function, followed by a natural log transformation and adding a pseudocount of 1. Highly variable genes were estimated using scanpy’s ‘highly_variable_genes’ function, after which a principal component analysis was run using the highly variable genes. A kNN graph was estimated from the top 50 principal components using k=15 nearest neighbors based on the UMAP neighborhood selection approach.^49^ Following this, Louvain clustering^50^ was performed using a resolution parameter of 1.5. Clusters exhibiting high expression levels of G2M cell cycle genes were subsequently filtered, as well as clusters with a subpallium (ventral cortical) identity, hippocampal identity, and Cajal-Retzius neurons. The above procedure was re-run until the only subsequent populations in the sample consisted of forebrain dorsal NSCs, IP cells, or neurons based on the expression of known marker genes for the respective populations. Diffusion pseudotime estimates^9^ for each cell were then estimated was after running a diffusion map embedding and assigning a starting cell. The raw count data across all cells ordered by diffusion pseudotime were then stored and the MCMC procedure was run on the resulting count matrix.

#### Processing scRNA-Seq of mouse e14.5 pancreas development samples

The raw count data for the pancreas endocrinogenesis dataset^46^ was downloaded from http://www.ncbi.nlm.nih.gov/geo/query/acc.cgi?acc=GSE132188. The raw count data was loaded into scanpy^47^ for downstream analyses. Cells were then initially subset to samples corresponding to e14.5 embryos. All cells with a positive G2M score in the metadata were initially filtered. Following this, the count data was processed in a similar fashion to the Atlas of the Developing Mouse Brain dataset using scanpy. Diffusion pseudotime estimates were calculated and the raw count data across all cells ordered by diffusion pseudotime were then stored and the MCMC procedure was run on the resulting count matrix.

#### Establishing a likelihood model

The negative binomial distribution has been shown to accurately describe the count data generated in scRNA-Seq experiments without the need to account for zero-inflation resulting from “dropout” events.^51^ The probability mass function for the negative binomial distribution can be parameterized using the mean, $\mu \in R^{+}$, and dispersion parameter, $\varphi \in R^{+}$, with y∈N, as follows,

(Equation 2) $$p\left(y|\mu ,\varphi \right)=\left(\begin{array}{l} y+\varphi -1\\ y \end{array}\right){\left(\frac{\mu }{\mu +\varphi }\right)}^{y}{\left(\frac{\varphi }{\mu +\varphi }\right)}^{\varphi }\text{.}$$

The mean and variance of the random variable Y∼NB(μ,φ) which follows a negative binomial distribution is then E[Y]=μ and $\text{Var}\left[Y\right]=\mu +\frac{\mu^{2}}{\varphi }$. For a gene *g* with measured counts of ${\vec{Y}}_{g}={\left\{y_{gt}\right\}}_{t=1,..,N}$ along a pseudotime trajectory with fixed pseudotime-step interval, ${\vec{\mu }}_{g}={\left\{\mu_{gt}\right\}}_{t=1,\ldots ,N}$ and ${\vec{\varphi }}_{g}={\left\{\varphi_{gt}\right\}}_{t=1,\ldots ,N}$ the mean and dispersion at corresponding pseudotimes, the full likelihood of observing ${\vec{Y}}_{g}$ is:

(Equation 3) $$L\left({\vec{\mu }}_{g}|{\vec{Y}}_{g},{\vec{\varphi }}_{g}\right)=\prod_{t=1}^{N}p\left(y_{gt}|\mu_{gt},\varphi_{gt}\right)\text{,}$$

where $p\left(y_{gt}|\mu_{gt},\varphi_{gt}\right)$ is the negative binomial probability mass function. The full log-likelihood is then:

(Equation 4) $$\ln\left(L\left({\vec{\mu }}_{g}|{\vec{Y}}_{g},{\vec{\varphi }}_{g}\right)\right)=\sum_{t=1}^{N}\ln\left(p\left(y_{gt}|\mu_{gt},\varphi_{gt}\right)\right)\text{.}$$

It was shown that when fitting scRNA-Seq UMI count data to a negative binomial model, data are consistent with a global dispersion parameter independent of the expression level of a given gene, and that fitting a dispersion parameter to each gene individually leads to overfitting.^52^ Therefore, a global estimate of φ can be used for every gene independent of pseudotime, and $\vec{\varphi_{g}}={\left\{\varphi_{gt}\right\}}_{t=1,\ldots ,N}$ is replaced with a constant φ in Equation 4. A dataset specific φ using genes which exhibit lower levels of overdispersion is estimated, since the expression levels in these genes reflect the technical rather than the biological variability. To do this, the log10 mean counts for each gene are binned into five equally spaced bins, and a linear fit between log10 mean and log10 variance of counts in each bin is estimated. Genes within the top 20th percentile of the difference between the estimated variance and the expected variance using the linear fit in each bin are then filtered. The remaining genes are used to fit the non-linear relationship between the mean (μ) and variance ($\sigma^{2}=\mu +\frac{\mu^{2}}{\varphi }$) using unconstrained non-linear least squares (Figure S9).

Here, φ estimates the dispersion based on genes which do not exhibit high variability in the dataset, and therefore captures the technical variability in the dataset. This technical variability is in large part driven by the varying number of UMI counts captured in each cell, as well as other factors including library quality and amplification bias. Thus, the full log-likelihood of observing counts ${\vec{Y}}_{g}={\left\{y_{gt}\right\}}_{t=1,..,N}$ for gene *g* along a pseudotime trajectory given the mean at corresponding pseudotime points ${\vec{\mu }}_{g}={\left\{\mu_{gt}\right\}}_{t=1,\ldots ,N}$, becomes,

(Equation 5) $$\ln\left(L\left({\vec{\mu }}_{g}|{\vec{Y}}_{g},\varphi \right)\right)=\sum_{t=1}^{N}\ln\left(p\left(y_{gt}|\mu_{gt},\varphi \right)\right)\text{,}$$

where φ is a global parameter estimated using the procedure described above.

For scRNA-Seq methods which sequence only from one end of the transcript and not full-length protocols, normalization does not need to account for the total transcript length. In this case, for a given cell *i*, let $M_{i}$ be the number of UMIs in cell *i*, and $y_{gi}$ be the number of UMIs for gene *g* in cell *i*. In this paper, we use the median number of UMIs across all cells in the dataset as a size factor $\tilde{M}$, that is, $\tilde{M}=\text{med}{\left\{M_{i}\right\}}_{i=1,\ldots ,N}$. Then, the log-normalized expression levels for gene *g* in cell *i* is defined by the following mapping,

(Equation 6) $$h\left(y_{gi}\right)={\tilde{y}}_{gi}=\ln\left(\frac{y_{gi}}{M_{i}}\tilde{M}+1\right)\text{.}$$

The functions $\left(f_{\text{unif}},f_{\text{gauss}},f_{\text{sig}},f_{\text{dsig}}\right)$ described in Equation 1 are then fit to the pseudotemporally ordered expression profile for gene *g*, ${\left\{{\tilde{y}}_{gt}\right\}}_{t=1,\ldots ,N}$, in the log-normalized expression space with the objective function to maximize defined by the likelihood in Equation 5. The means ${\vec{\mu }}_{g}={\left\{\mu_{gt}\right\}}_{t=1,\ldots ,N}$ are then calculated by mapping the function values evaluated at t=1,…,N back to count space using the inverse of Equation 6. The full log-likelihood estimate is then evaluated by plugging in the ${\vec{\mu }}_{g}$ values and global estimate for φ into Equation 5.

This procedure can be summarized as follows. We want to solve for $f_{\alpha }\left(t;\theta \right)$, which maximizes the following likelihood,

(Equation 7) $$\ln\left(L\left({\vec{\mu }}_{g}|{\vec{Y}}_{g},\varphi \right)\right)=\sum_{t=1}^{N}\ln\left(p\left(y_{gt}|h^{-1}\left(f_{\alpha }\left(t;\theta \right)\right),\varphi \right)\right)\text{,}$$

where $f_{\alpha }\in \left(f_{\text{unif}},f_{\text{gauss}},f_{\text{sig}},f_{\text{dsig}}\right)$.

#### Model inference using MCMC

Under the framework presented above, solving for $f_{\alpha }\left(t;\theta \right))$ can be formulated as a Bayesian inference problem, which we solve using an ensemble sampler MCMC approach.^19^ This provides an estimate of the posterior distribution over the parameter space for each of the parameters in the different functions $\left(f_{\text{unif}},f_{\text{gauss}},f_{\text{sig}},f_{\text{dsig}}\right)$ described in Equation 1. For each of the models, the priors used for the different parameters are summarized in Table S4.

Note, in Table S4, the folded normal distribution is parameterized by μ>0 and σ>0 with probability density function,

(Equation 8) $$p\left(x;\mu ,\sigma^{2}\right)=\frac{1}{\sqrt{2\pi \sigma^{2}}}e^{-\frac{{\left(x-\mu \right)}^{2}}{2\sigma^{2}}}+\frac{1}{\sqrt{2\pi \sigma^{2}}}e^{-\frac{{\left(x+\mu \right)}^{2}}{2\sigma^{2}}}\text{.}$$

The uniform priors in Table S4 are uninformative, however, they provide bounds on the parameters to keep them in interpretable and meaningful ranges. The slope parameters *k* in the sigmoidal function, and $k_{1}$ and $k_{2}$ in the double sigmoidal function, have a folded normal prior with 0-mean and 0.1 variance, which is used to ensure that the slope has a low magnitude. This prior is used because differences in the function once the slope becomes relatively large are minimal. Finally, the folded normal prior on σ in the Gaussian with 0-mean and N/10 variance is used to ensure that the curve does not become very flat.

In this paper, we use the ensemble sampler MCMC proposed by Goodman & Weare in 2010^19^ with implementation by Foreman–Mackey et al.^53^ An initial guess is needed as a starting point from which a walker begins in the ensemble sampler. For the Gaussian and sigmoidal functions, initial guesses are derived from a non-linear least squares fit for each function on the log-normalized pseudotime expression levels using scipy’s ‘curve_fit’ function, with added Gaussian noise. For the double sigmoidal function, initial guesses are randomly chosen to cover the varieties of different forms the functions can have. For the uniform function, initial guesses are randomly chosen from a uniform distribution over the interval 0.01 and maximum expression level for the gene of interest. The number of walkers used is four times the number of parameters for each function — 28 for the double sigmoidal fit, 16 for the Gaussian fit, 16 for the sigmoidal fit, and 4 for the uniform fit. This enables a wide sampling across the search space of parameters.

The MCMC is then run for a total of 10,000 iterations. There is generally no consensus on how many iterations to run an MCMC algorithm.^53^ Thousands of iterations are typically desirable to allow the process to reach a steady-state. After reaching the steady-state, the MCMC will sample from the posterior distribution over the parameter space, enabling an estimate of the posterior distribution for each parameter. Iterations before reaching the steady-state are discarded, as these are not sampled from the target distribution. This is called the “burn-in” phase. For this implementation, a burn-in of 5,000 iterations was used (Figure S10).

Some MCMC walkers can get stuck near a local maximum. These walkers typically have a low acceptance rate, that is the proportion of moves for which the MCMC sampler generated parameter values that differed from the previous sample. One common practice is to prune these walkers from the final MCMC output. For example, walkers can be pruned which get stuck in irrelevant local optima by clustering the likelihood of the walkers and removing the clusters with lower likelihoods.^54^ For this implementation, half of the MCMC walkers are pruned with the lowest acceptance rate in order to remove potentially stuck walkers (Figure S11).

#### Model selection

We use a probabilistic model selection technique, the bayesian information criterion (BIC)^55^ to score the different models, and select the model with the best score. The BIC is defined as follows,

(Equation 9) $$\text{BIC}=k\;\ln\left(n\right)-2\;\ln\left(\hat{L}\right)\text{,}$$

where n= number of data points, k= number of parameters in the model, and $\hat{L}=$ maximized value of the likelihood function. In the original formulation of the BIC, the value $\hat{L}$ was derived from maximum likelihood estimation. When using an MCMC for model inference, the output consists of a sampling or distribution over the parameter space. It is advantageous to use a likelihood estimate which more closely reflects the optimal parameter regime estimated from the MCMC instead of the parameter regime which maximizes the likelihood. To this end, $\hat{L}$ in the BIC equation in Equation 9 is replaced with P(y|⟨θ⟩), the likelihood of observing the data given ⟨θ⟩, where ⟨θ⟩= mean over the parameter estimates across all MCMC iterations.

To improve the generalizability of a model fit to the dataset, and remove the bias of outliers, we developed a variation of cross-validation for model selection, described in Algorithm 1.

Algorithm 1 **Perform model selection based on MCMC runs**

1: Measure average parameter estimates, ⟨θ⟩, across MCMC runs for each model.

2: Remove 2% of the data chosen randomly ($y_{sub}$), and estimate BIC for each model using $P\left(y_{sub}|\left\langle \theta \right\rangle \right)$.

3: Repeat Step 2. for 10,000 subsets. Define ${\text{BIC}}_{x}$ as the set of BIC estimates across all 10,000 subsets for a given fit, and $\left\langle {\text{BIC}}_{x}\right\rangle$ as the mean BIC estimate across all 10,000 subsets.

4: **if** max(${\text{BIC}}_{\text{double}\;\text{sigmoidal}}$) < min(${\text{BIC}}_{\text{uniform}}$) & $\left\langle {\text{BIC}}_{\text{double}\;\text{sigmoidal}}\right\rangle <\left\langle {\text{BIC}}_{\text{gauss}}\right\rangle$ & $\left\langle {\text{BIC}}_{\text{double}\;\text{sigmoidal}}\right\rangle <\left\langle {\text{BIC}}_{\text{sigmoidal}}\right\rangle$ **then**

5:  Set best fit to double sigmoidal.

6: **else if** max(${\text{BIC}}_{\text{sigmoidal}}$) < min(${\text{BIC}}_{\text{uniform}}$) & $\left\langle {\text{BIC}}_{\text{sigmoidal}}\right\rangle <\left\langle {\text{BIC}}_{\text{gauss}}\right\rangle$ **then**

7:  Set best fit to sigmoidal.

8: **else if** max(${\text{BIC}}_{\text{gauss}}$) < min(${\text{BIC}}_{\text{uniform}}$) & $\left\langle {\text{BIC}}_{\text{gauss}}\right\rangle <\left\langle {\text{BIC}}_{\text{sigmoidal}}\right\rangle$ **then**

9:  Set best fit to Gaussian.

10: **else**.

11:  Set best fit to uniform.

12: **end if**.

Note, instead of cross-validating a model estimated from a training set on a test set, the full dataset is used for model inference and tested on random subsets of the dataset. Figure S12 highlights a random sampling of the parameters over the MCMC runs using a double sigmoidal, Gaussian, sigmoidal and uniform model, as well the BIC estimates on random 98% subsets of the data.

It is worth noting that the double sigmoidal function can also closely take the form of the Gaussian and sigmoidal functions. It would be possible to use the double sigmoidal function alone, instead of including the Gaussian and sigmoidal functions, to model the dynamics of gene expression. However, the double sigmoidal function will force the presence of two inflection points, whereas with the sigmoidal function will only have one inflection point, which in many cases more accurately models the gene expression dynamics of single a state-switch. Finally, a simpler model is often more favorable to use than a more complex model to prevent overfitting, and in the cases where a Gaussian function provides an equally good fit as the double sigmoidal function, then the selection of the simpler Gaussian model is preferred.

#### MCMC diagnostics

In order to ensure that the MCMC adequately approximates the posterior distribution over the parameter space, a variety of heuristics exist. The MCMC trace plot (Figure S10) provides a visual inspection of whether the MCMC appears to have reached a steady-state. Also, the acceptance fraction across MCMC chains (Figure S11) is used to filter potentially stuck MCMC walkers. In general, there is no way to prove convergence of an MCMC sampler,^56^ and therefore diagnostics are used to measure how well an MCMC run has converged to an equilibrium or steady-state. A few diagnostics are highlighted in this section to show the ability of the ensemble sampler described above to adequately converge to the posterior distribution over the parameter space.

One diagnostic metric relies on the estimate of the integrated autocorrelation time, which estimates the number of iterations needed for the MCMC to draw an independent sample. In the case of samples generated by an MCMC, the samples are not independent. This is due to the nature of the Markov process used to sample from the posterior distribution, which is dependent on the previous sampling of parameters, by definition. The integrated autocorrelation time is defined as,

(Equation 10) $$\tau_{f}=\sum_{\tau =-\infty }^{\infty }\rho_{f}\left(\tau \right)=1+2\sum_{\tau =1}^{\infty }\rho_{f}\text{,}$$

where $\rho_{f}\left(\tau \right)$ is the autocorrelation function at time delay τ. Then, the effective sample size (ESS), i.e. the number of i.i.d. draws from the posterior distribution, for an ensemble sampler can be calculated as,

(Equation 11) $$\text{ESS}=\frac{MN}{\tau_{f}}\text{,}$$

where M= number of walkers, and N= number of MCMC iterations used after discarding the burn-in. In order to estimate $\tau_{f}$, the marginal autocorrelation function for each parameter in the model can be estimated separately out to a certain time delay, *T*, using the average estimate across all walkers, and taking the maximum estimate of $\tau_{f}$ over all *T*, defined as

(Equation 12) $${\hat{\tau }}_{f}=\max_{T}\left(1+2\sum_{\tau =1}^{T}<\rho_{f}\left(\tau \right)>\right)\text{.}$$

Here, T∈[0,1000] enables an accurate estimate of ${\hat{\tau }}_{f}$ under the assumption that $\rho_{f}\left(\tau \right)$ approaches 0 by τ=T for each parameter. The autocorrelation function (Figure S13) and autocorrelation time (Figure S14) is estimated for each parameter separately.

For a general comparison, the autocorrelation times were estimated for all genes using the model with the best fit in the mouse e13.5 forebrain sample (Figure S15). The autocorrelation times increase with the complexity of the model (i.e. number of parameters specified in each model). This is in part expected, since a model with more parameters will generally have a lower acceptance rate due to the higher number of dimensions in which the MCMC has to make proposal moves, leading to higher autocorrelations for each parameter. Nonetheless, the autocorrelation times are fairly robust for each model.

Thinning is an approach to use every *k*-th iteration of the MCMC walkers, where $k=\tau_{f}$ would represent an i.i.d. sampling of the posterior distribution. However, various publications indicate that thinning is often unnecessary and results in reduced precision.^57^^,^^58^ Therefore, no thinning of the MCMC walkers was used in this analysis.

Another way to visualize the posterior distribution over the parameter space derived from an MCMC is a corner plot (Figure S16). The corner plot highlights both the two dimensional projections over the parameter space across iterations of the MCMC, as well as the marginal posterior distribution for each individual parameter (highlighted in the upper plots). Some parameters are more correlated with each other than others, indicating underlying covariates within the model parameters. However, the marginal posterior distributions do not appear to be multimodal.

These heuristics provide some insight into the ability of the ensemble MCMC sampler to provide an accurate sampling of the posterior distribution over the parameter space.

#### Estimating inflection points

Inflection points occur where the curvature of a function changes sign. At inflection points, the first-order derivative, or rate of change, of a function reaches a local maximum or local minimum. At an inflection point, the second-derivative of a function passes through 0 with the second derivative changing sign from positive (concave upward) to negative (concave downward) or vice versa. The inflection points of the Gaussian, sigmoidal and double sigmoidal fits can be used to compare the relative timing of when genes exhibit a state transition along a pseudotime trajectory. To estimate the inflection points of the different functions, first solve for *x* at which the second-derivative of the function is zero. For the Gaussian function, $f_{\text{gauss}}\left(t\right)$, sigmoidal function $f_{\text{sig}}\left(t\right)$, and double simgoidal function $f_{\text{dsig}}\left(t\right)$ defined in Equation 1, the second derivatives are

$$f_{\text{gauss}}^{\prime \prime }\left(t\right)=\frac{a}{\sigma^{4}}e^{-\frac{{\left(t-t_{0}\right)}^{2}}{2\sigma^{2}}}\left(t-\left(t_{0}-\sigma \right)\right)\left(t-\left(t_{0}+\sigma \right)\right)\text{,}$$

$$f_{\text{sig}}^{\prime \prime }\left(t\right)=k^{2}L\frac{e^{-k\left(t-t_{0}\right)}\left(e^{-k\left(t-t_{0}\right)}-1\right)}{{\left(1+e^{-k\left(t-t_{0}\right)}\right)}^{3}}\text{,}$$

$$f_{\text{dsig}}^{\prime \prime }\left(t\right)=k_{1}^{2}\left(b_{mid}-b_{\min}\right)\frac{e^{-k_{1}\left(t-t_{1}\right)}\left(e^{-k_{1}\left(t-t_{1}\right)}-1\right)}{{\left(1+e^{-k_{1}\left(t-t_{1}\right)}\right)}^{3}}+k_{2}^{2}\left(b_{\max}-b_{mid}\right)\frac{e^{-k_{2}\left(t-t_{2}\right)}\left(e^{-k_{2}\left(t-t_{2}\right)}-1\right)}{{\left(1+e^{-k_{2}\left(t-t_{2}\right)}\right)}^{3}}\text{.}$$

For the Gaussian function, $f_{\text{gauss}}\left(t\right)$, two inflection points occur at $t\in \left(t_{0}-\sigma ,t_{0}+\sigma \right)$. For the sigmoidal function, $f_{\text{sig}}\left(t\right)$, one inflection point occurs at $t=t_{0}$. The estimates for the inflection points are then measured from the parameters $\left(t_{0}-\sigma ,t_{0}+\sigma \right)$ for the case of the Gaussian and $t_{0}$ for the case of sigmoidal function at each MCMC iteration. Finally, for the double sigmoidal function, $f_{\text{dsig}}\left(t\right)$, the number of inflection points can vary. However, if all parameters are fixed besides $k_{1}$, then, $f_{\text{dsig}}^{\prime \prime }\left(t\right)\to 0$ as $k_{1}$ increases. Similarly, if all parameters are fixed besides $t_{1}$, then $f_{\text{dsig}}^{\prime \prime }\left(t\right)\to 0$ as $t_{1}$ decreases. That is, for $k_{1}\gg 0$, i.e. the transition from $b_{min}$ to $b_{mid}$ occurs rapidly, then an inflection point will occur very close to $t_{1}$. Similarly, for $k_{2}\gg 0$, i.e. the transition from $b_{mid}$ to $b_{\max}$ occurs rapidly, then an inflection point will occur very close to $t_{2}$. Also, the further apart $t_{1}$ and $t_{2}$ are from each other, the closer the inflection points are to $t_{1}$ and $t_{2}$. To ensure the inflection points occur very close to $t_{1}$ and $t_{2}$, at each iteration of the MCMC, a move is only accepted in cases where $\text{sign}\left(f_{\text{dsig}}^{\prime \prime }\left(t_{1}-dt\right)\right)*\text{sign}\left(f_{\text{dsig}}^{\prime \prime }\left(t_{1}+dt\right)\right)<0$ and $\text{sign}\left(f_{\text{dsig}}^{\prime \prime }\left(t_{2}-dt\right)\right)*\text{sign}\left(f_{\text{dsig}}^{\prime \prime }\left(t_{2}+dt\right)\right)<0$ for dt=1. The estimates for the inflection points are then calculated from the parameters $t_{1}$ and $t_{2}$ at each MCMC iteration.

#### Comparing inflection points

Regulatory interactions were inferred based on the relative timing of inflection point estimates (Figure 2). If there was an overlap of at least 1% in the inflection point estimates between two genes across MCMC iterations, then these were assumed to have a simultaneous switch state. A regulatory interaction between the two was mutually positive if the inflection points had the same sign, and mutually negative if the inflection points differed in sign. The overlap between two inflection points is estimated by binning the inflection point estimates across all MCMC iterations to 100 equally spaced bins, starting at the minimum inflection point estimate across both genes and ending at the maximum inflection point estimate across both genes. Let ${\left\{x_{i}\right\}}_{i\in \left[1,100\right]}$ represent this binning domain. If $p_{A}\left(x_{i}\right)$ is the percent of counts in the histogram in bin $x_{i}$ for gene A, and $p_{B}\left(x_{i}\right)$ is the percent of counts in the histogram in bin $x_{i}$ for gene B, then the overlap between the two, P(A=B), is

(Equation 13) $$P\left(A=B\right)=\sum_{i=1}^{100}\min\left(p_{A}\left(x_{i}\right),p_{B}\left(x_{i}\right)\right)\text{.}$$

If the inflection point estimates were non-overlapping (*i.e.* inflection point overlap was less than 1%), then the following relationships were constructed. If the first gene (*i.e.* earlier inflection point) had a positive inflection point and the second gene (*i.e.* later inflection point) also had a positive inflection point, then the first gene positively regulates the second gene. If the first gene had a positive inflection point and the second gene had a negative inflection point, then the first gene negatively regulates the second gene, and the second gene positively regulates the first. If the first gene had a negative inflection point and the second gene had a negative inflection point, then the first gene positively regulates the second gene. If the first gene had a negative inflection point and the second gene had a positive inflection point, then no relationship is given.

#### Running Monocle 3, tradeSeq and Slingshot on mouse e13.5 forebrain dorsal cells

We tested whether genes were differentially expressed along the e13.5 forebrain dorsal NSC → IP → neuron trajectory using Monocle 3 and tradeSeq. tradeSeq works by fitting a negative binomial generalized additive model (GAM) to the pseudotime-ordered counts for each gene separately. We used the associationTest() from tradeSeq, which tests the null hypothesis that all smoother coefficients in the GAM are equal to each other. We passed in the raw counts and pseudotime ordering from diffusion pseudotime as input, specifying the number of knots used for the GAM fitting to 3. To test whether genes were differentially expressed along the trajectory using Monocle 3, we used the graph_test() function, passing in the principal_graph estimated by the learn_graph() function, which estimates a pseudotime trajectory by fitting a principal graph through the cells. Finally, to compare the affect of input pseudotime method, we also estimated a pseudotime ordering of e13.5 forebrain dorsal NSC → IP → neurons using Slingshot, which fits a principal curve through the data. As input, we passed in the 2-dimensional umap embedding of these cells and log-normalized expression data, using the getLineages() function to estimate the pseudotime ordering.

## Supplemental information

Document S1. Figures S1–S16 and Table S4

Table S1. Best fits and inflection point estimates mouse e13.5 forebrain dorsal NSC → IP → neurons sample 1, related to Figures 1 and 2

Table S2. Best fits and inflection point estimates mouse e13.5 forebrain dorsal NSC → IP → neurons sample 2, related to Figure 3

Table S3. Best fits and inflection point estimates mouse e14.5 pancreatic beta cell development, related to Figure 5
