# Supplementary material for: Modeling gene expression cascades during cell state transitions
Source: iScience. 2024 Mar 4;27(4):109386. doi: 10.1016/j.isci.2024.109386 (PMC10946328; doi:10.1016/j.isci.2024.109386)
Supplement: Document S1. Figures S1–S16 and Table S4 [file mmc1.pdf]

iScience, Volume *27*

Supplemental information

Modeling gene expression cascades during cell state transitions

Daniel Rosebrock, Martin Vingron, and Peter F. Arndt

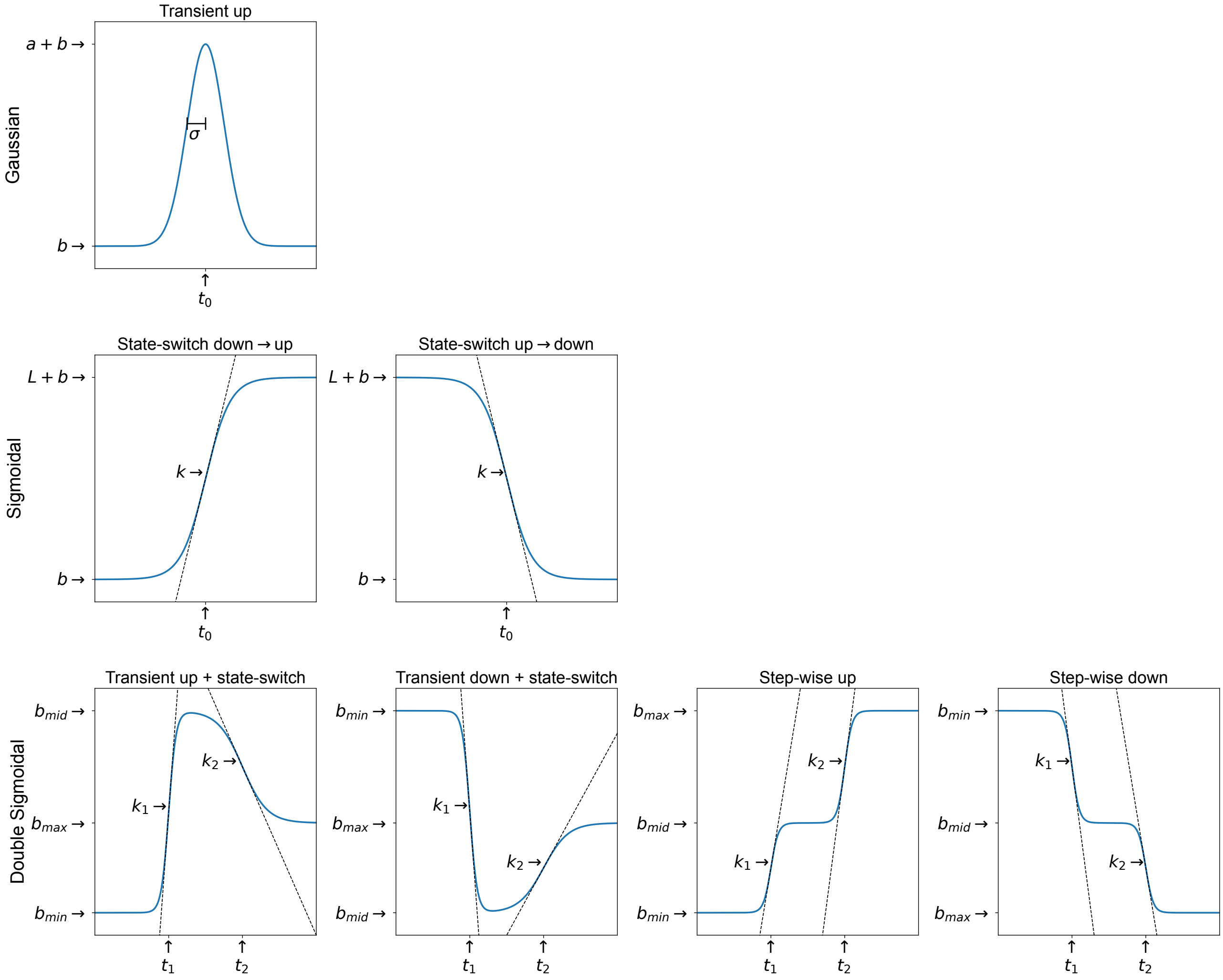

Figure S1: Modeling gene expression dynamics, Related to Equation 1 and STAR Methods. Gaussian, sigmoidal, and double sigmoidal functions can be used to model a variety of expression dynamics along differentiation trajectories. The plots highlight the different expression dynamics which can be modeled with each function. For the Gaussian function, t_0 specifies the location of the peak, b the basal expression level, $a + b$ the peak expression level, and σ the standard deviation. For the sigmoidal function, t_0 specifies the location of the inflection point, b the basal expression level, $L + b$ the peak expression level, and k the rate of increase or decrease from basal to peak expression level. For the double sigmoidal function, t_1 and t_2 specify the location of the first and second inflection points, b_{min}, b_{mid} and b_{max} specify the initial, transitional, and final steady state expression levels, and k_1 and k_2 specify the rates of increase or decrease at each inflection point, or state change.

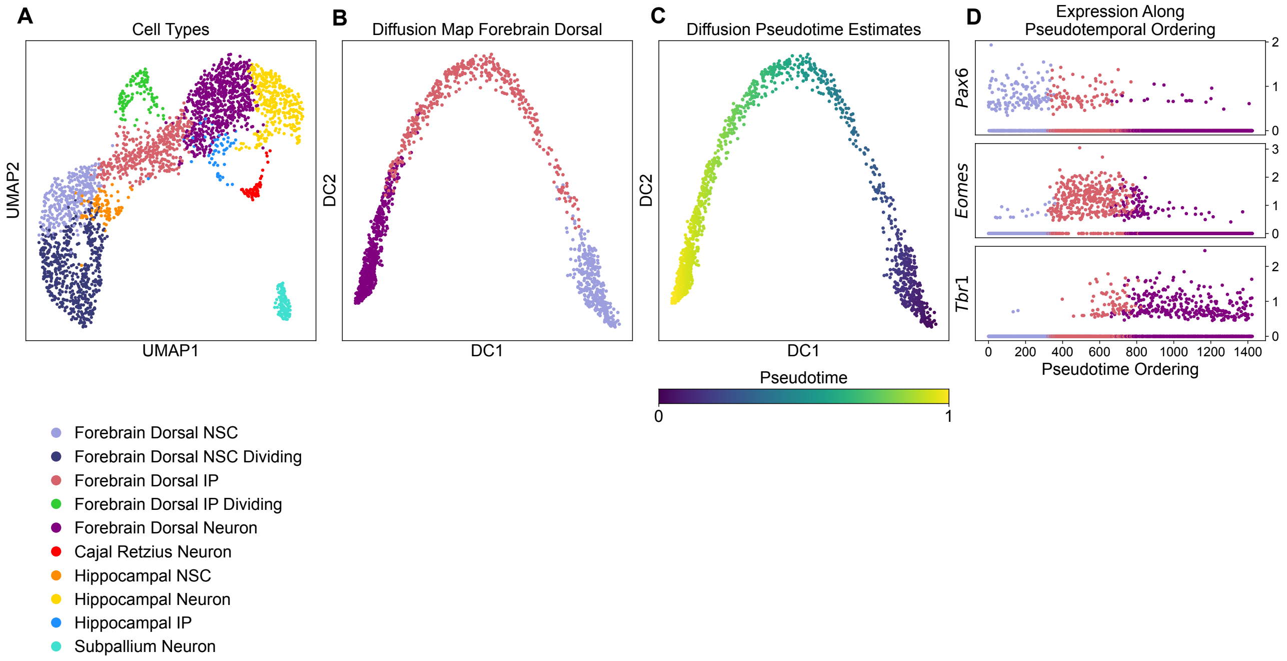

Figure S2: Ordering cells along forebrain dorsal differentiation trajectory in mouse e13.5 sample, Related to Figure 1 and Figure 2. (A) UMAP of cells in a scRNA-Seq experiment of a mouse e13.5 forebrain sample. (B) Diffusion map embedding of the forebrain dorsal NSC, IP and neuronal populations, after removal of all dividing cell types. (C) Diffusion map pseudotime estimates using the same embedding from (B). (D) Expression levels of key developmental transcription factors for cortical neurogenesis, *Pax6*, *Eomes* and *Tbr1*, within cells after ordering them according to their relative pseudotemporal ordering, with cells colored by their respective cell type annotations.

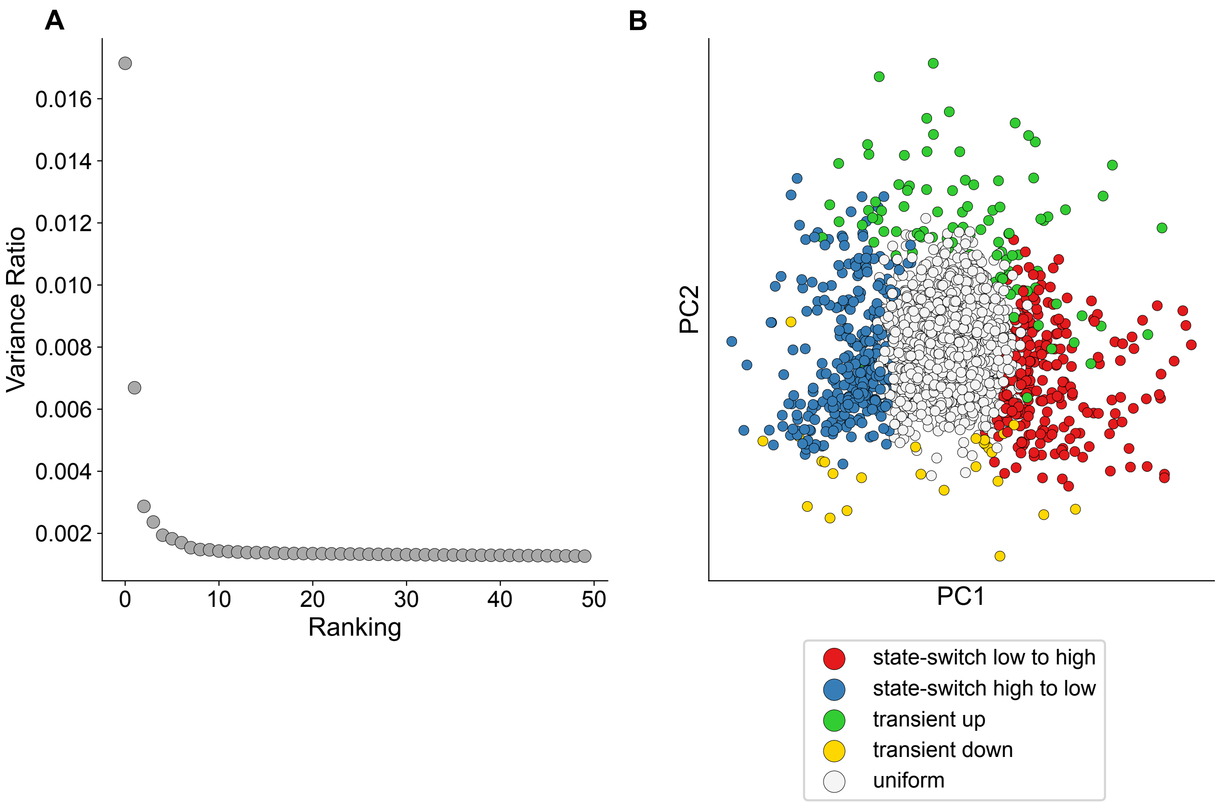

Figure S3: Functional forms capture the majority of variance in gene expression, Related to Figure 1. (A) A PCA was performed on the scaled expression levels of genes expressed in at least 1% of cells in the mouse e13.5 forebrain dorsal differentiation trajectory. The plot displays the variance ratio of each principal component of the top 50 ranked principal components. (B) The first two principal components are shown with the genes colored by their respective functional form: state-switch high to low (sigmoidal fit with negative inflection point or double sigmoidal fit with two negative inflection points), state-switch low to high (sigmoidal fit with positive inflection point or double sigmoidal fit with two positive inflection points), transient up (Gaussian or double sigmoidal fit), transient down (double sigmoidal fit), or uniform.

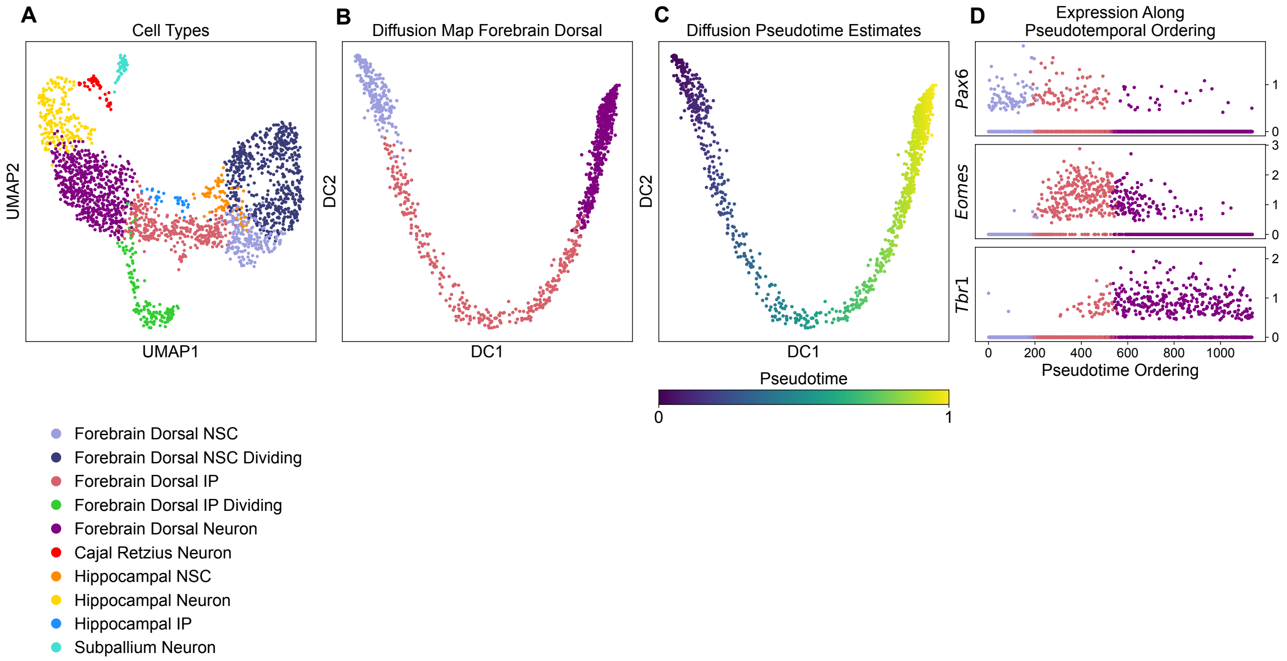

Figure S4: Ordering cells along forebrain dorsal differentiation trajectory in mouse e13.5 sample biological replicate, Related to Figure 3 and Figure 4. (A) UMAP of cells in a scRNA-Seq experiment of a mouse e13.5 forebrain sample. (B) Diffusion map embedding of the forebrain dorsal NSC, IP and neuronal populations, after removal of all dividing cell types. (C) Diffusion map pseudotime estimates using the same embedding from (B). (D) Expression levels of key developmental transcription factors for cortical neurogenesis, *Pax6*, *Eomes* and *Tbr1*, within cells after ordering them according to their relative pseudotemporal ordering using a fixed time step of 1, with cells colored by their respective cell type annotations.

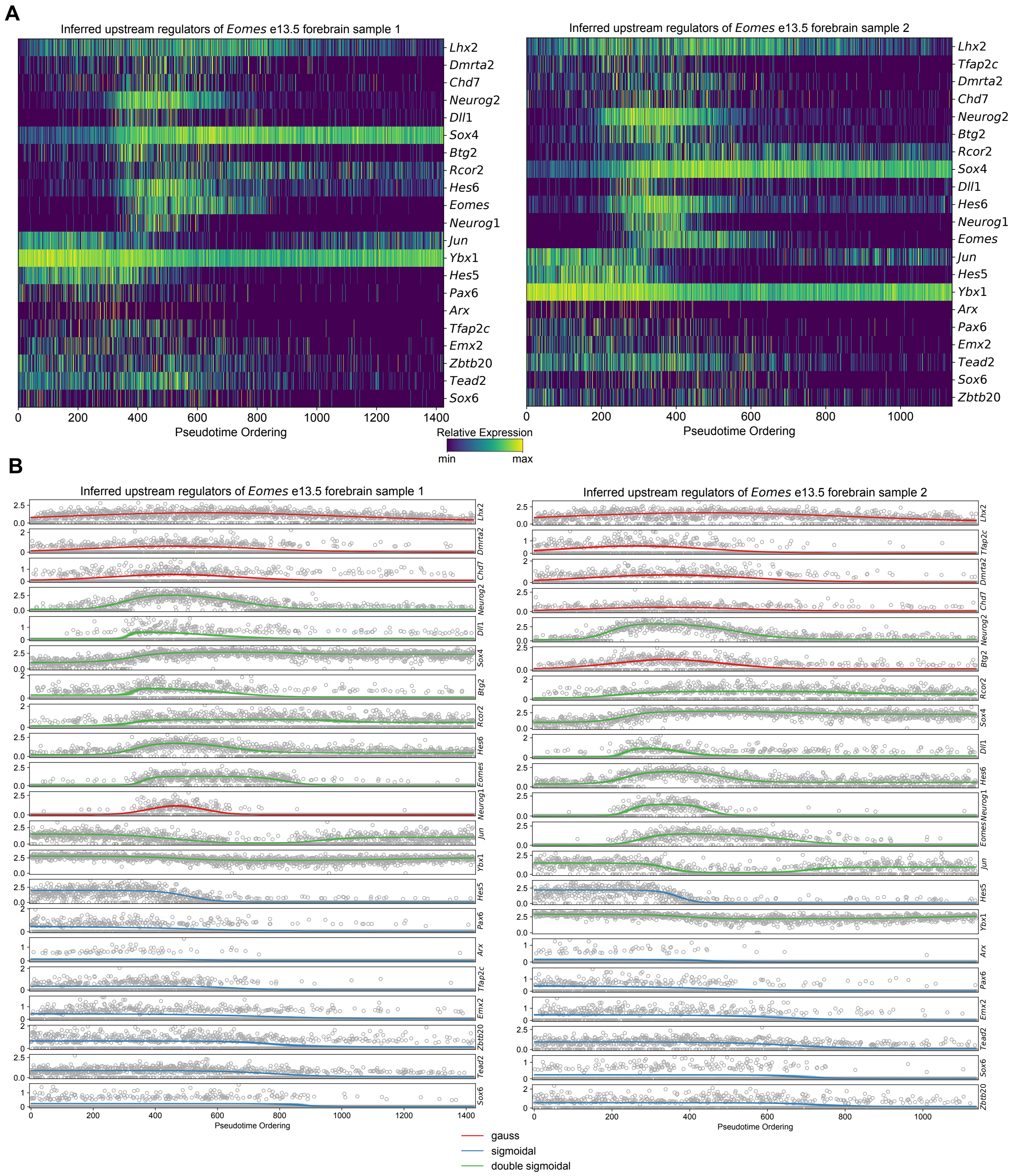

Figure S5: Potential upstream regulators of *Eomes* across mouse e13.5 embryos, Related to Figure 3. (A) Gene expression profiles of potential upstream regulators of *Eomes* measured across biological replicates in mouse e13.5 embryos are displayed as heatmaps in the left and right panel. (B) Gene expression profiles and best fitting model from our method of potential upstream regulators of *Eomes* measured across biological replicates in mouse e13.5 embryos are displayed in the left and right panel.

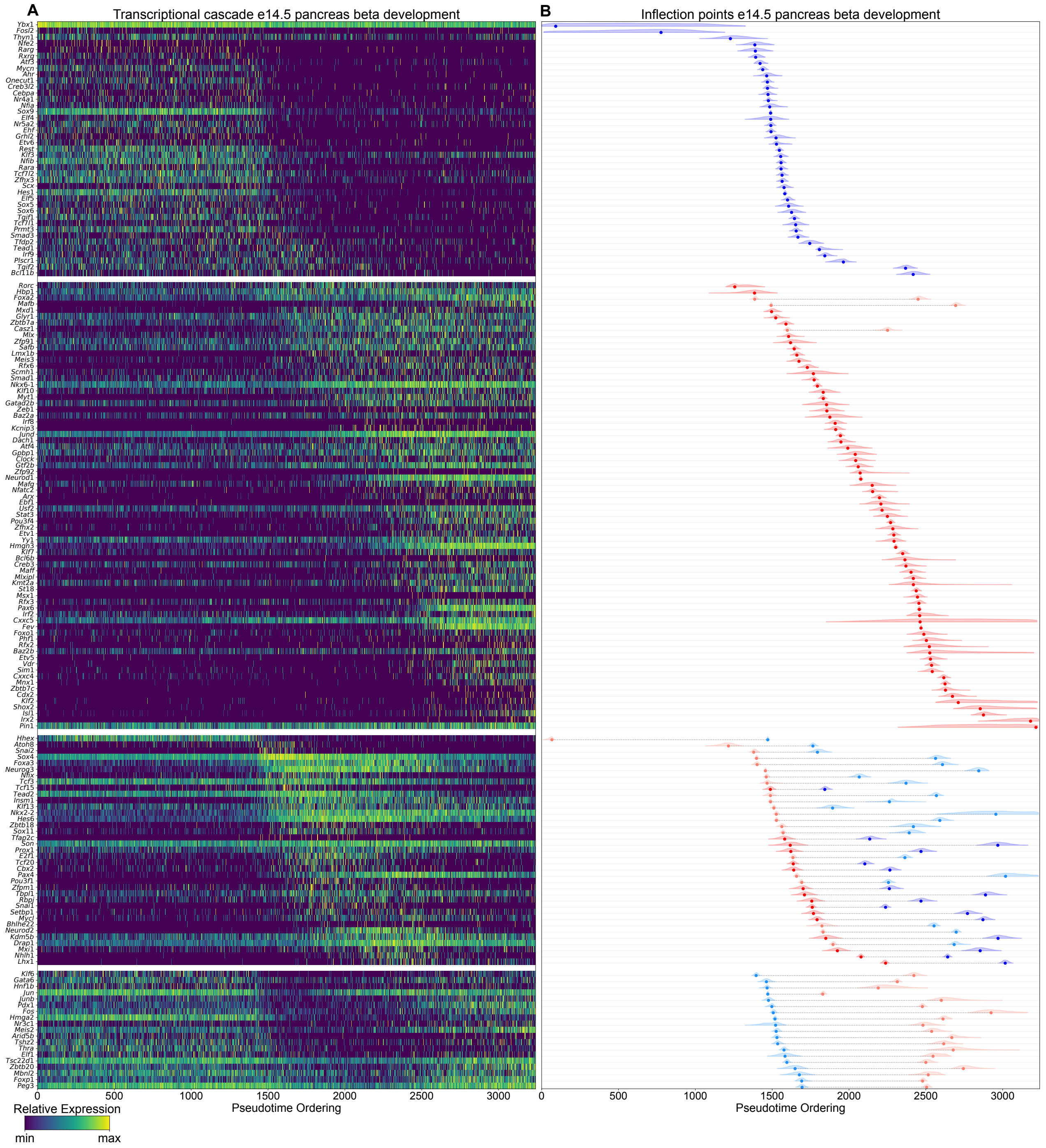

Figure S6: Transcriptional cascades in mouse e14.5 pancreatic beta cell development, Related to Figure 5. (A) Gene expression profiles of transcription factors with non-uniform fits are displayed as a heatmap. Genes are grouped according to a state-switch from high to low expression (sigmoidal fit) or step-wise down-regulation (double sigmoidal fit), state-switch from low to high expression (sigmoidal fit) or step-wise up-regulation (double sigmoidal fit), transient up (Gaussian or double sigmoidal fit), and transient down (double sigmoidal fit). (B) The inflection point estimates are shown from the same genes as in (A). Inflection point estimates from double sigmoidal fits are shown in light blue and light red, and those from Gaussian and sigmoidal fits in blue and red.

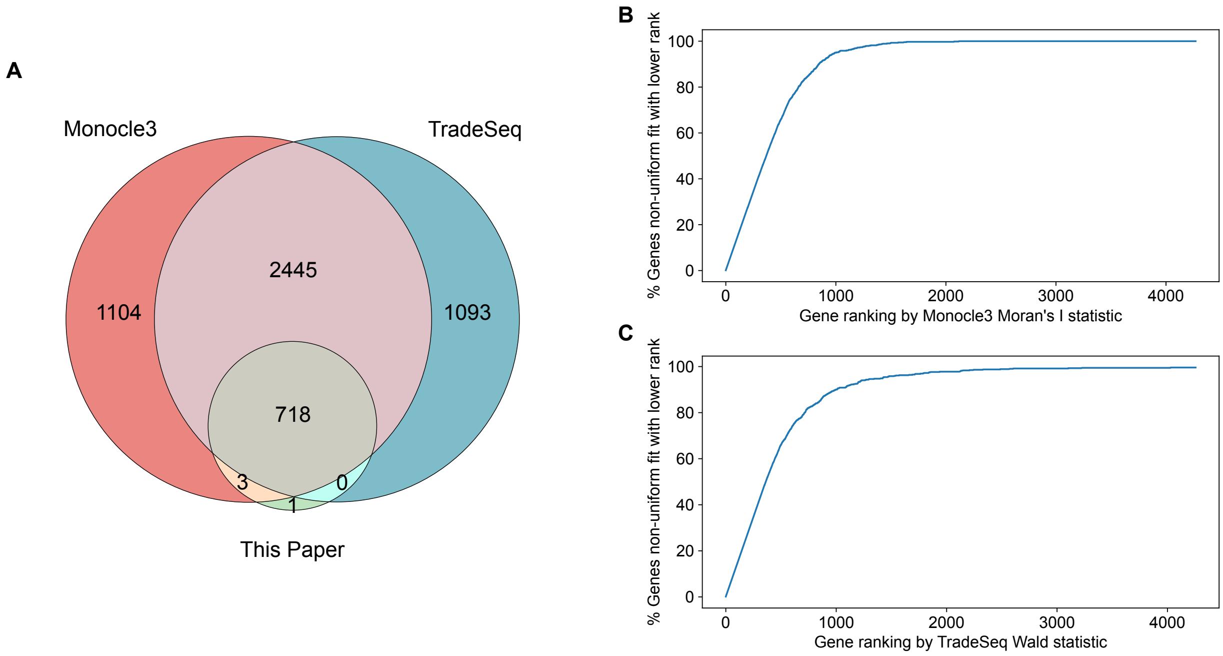

Figure S7: Comparison of dynamic genes with results from Monocle 3 and tradeSeq, Related to Figure 1. (A) The Venn diagram displays the genes with a non-uniform fit using our method compared with the significantly differentially expressed genes measured by Monocle 3 and tradeSeq in differentiating mouse e13.5 forebrain dorsal cells. (B) Genes were ordered by the Wald statistic measured by tradeSeq on the x-axis, and the percentage of genes with a non-uniform fit by our method with lower Wald statistic rank is plotted on the y-axis. (C) Genes were ordered by the Moran's I statistic measured by Monocle 3 on the x-axis, and the percentage of genes with a non-uniform fit by our method with lower Wald statistic rank is plotted on the y-axis. Notably, the majority genes with a non-uniform fit from our method have a lower ranking in both tradeSeq and Monocle 3.

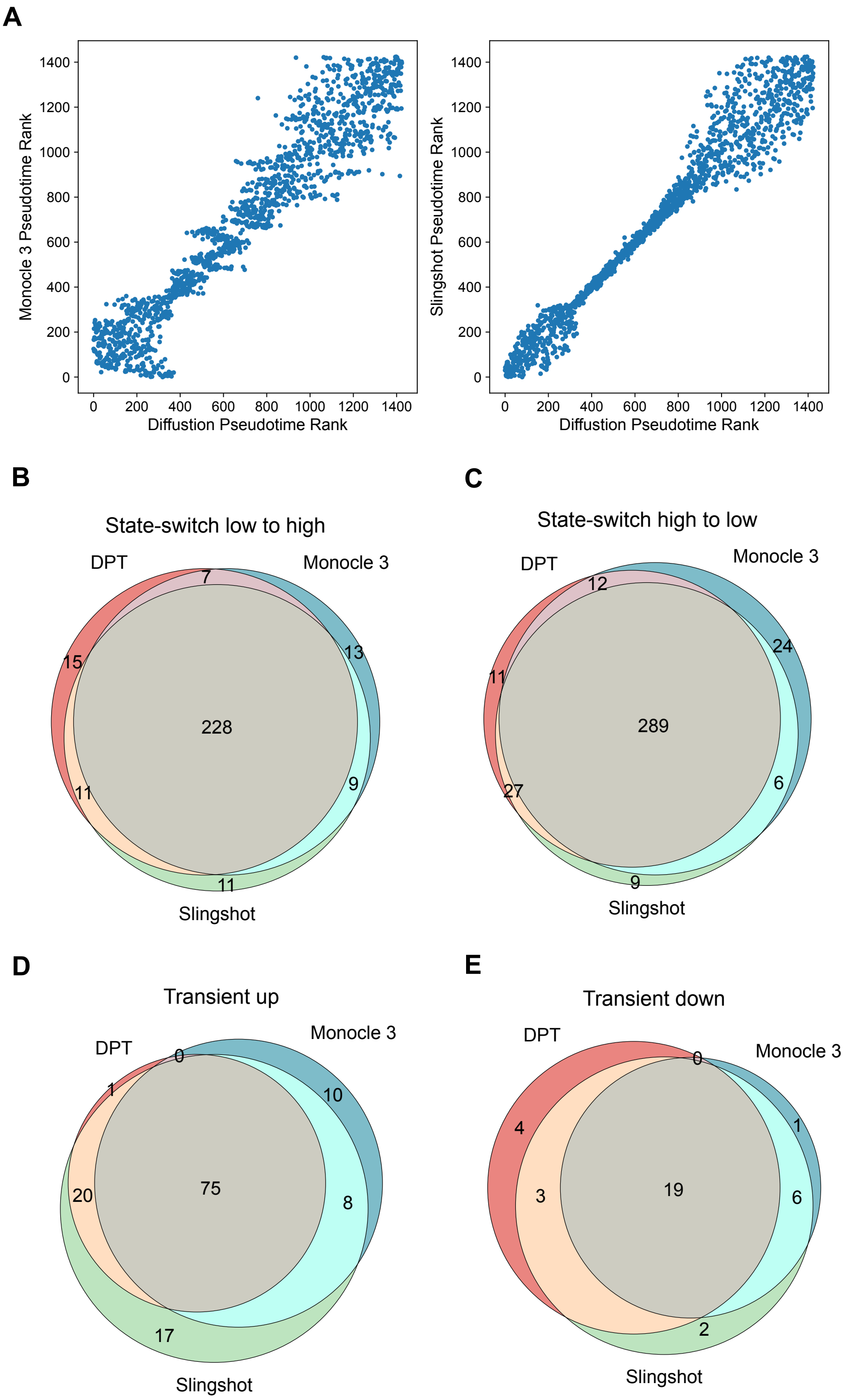

Figure S8: Comparison of best fits with different pseudotime methods, Related to Figure 1. (A) Comparison of diffusion pseudotime (DPT) ranks with Monocle 3 and Slingshot pseudotime ranks in differentiating mouse e13.5 forebrain dorsal cells. (B-E) The Venn diagrams display the genes with a state-switch from low to high (sigmoidal or double sigmoidal fit) (B), state-switch from high to low (sigmoidal or double sigmoidal fit) (C), transient up fit (Gaussian or double sigmoidal fit) (D), or transient down fit (Gaussian or double sigmoidal fit) (E), using DPT, Monocle 3, and Slingshot pseudotime estimates.

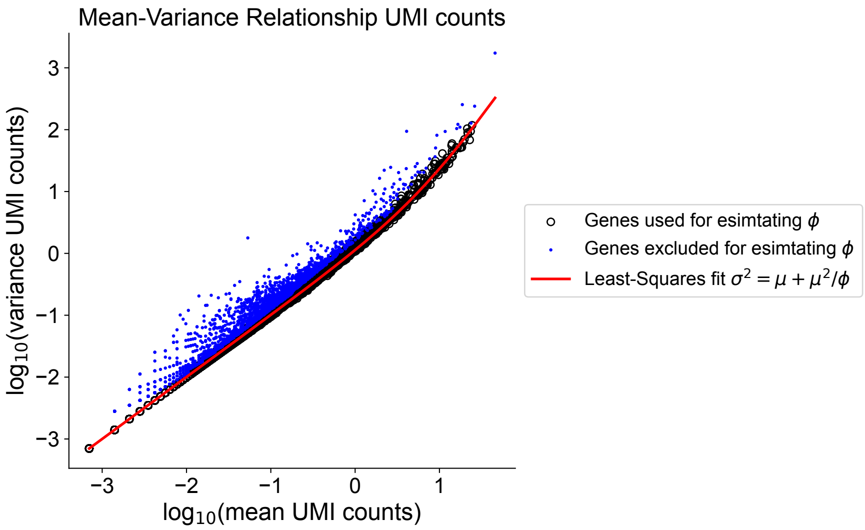

Figure S9: Estimating ϕ in scRNA-Seq UMI count data, Related to STAR Methods. A least-squares fit of the form $\sigma^2 = \mu + \frac{\mu^2}{\phi}$ in scRNA-Seq data of non-dividing mouse e13.5 forebrain dorsal cells. Fits are performed in the raw space, and are plotted after log10 transformation of both mean and variance of UMI counts.

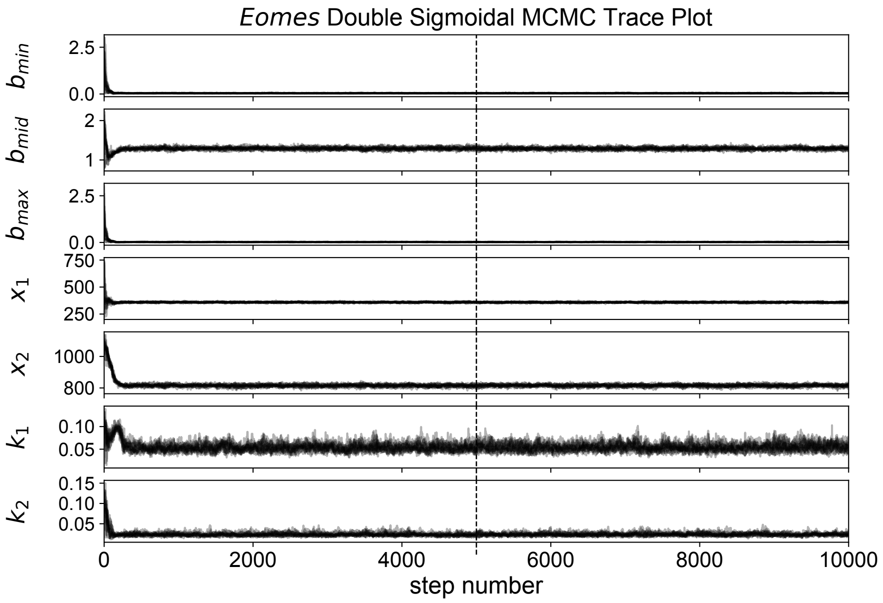

Figure S10: MCMC trace plot for *Eomes* in differentiating mouse e13.5 forebrain dorsal cells, Related to STAR Methods. MCMC trace plot for parameters $b_{min}, b_{mid}, b_{max}, x_1, x_2, k_1$, and k_2 in the double sigmoidal MCMC ensemble sampler for *Eomes*. The dashed line represents the burn-in time at 5,000 iterations. In this example, only 500 iterations are needed for the sampler to reach a steady-state.

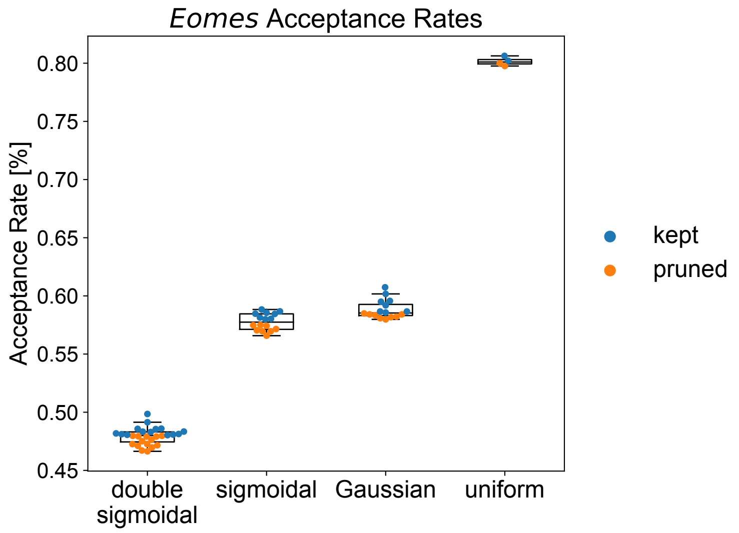

Figure S11: Acceptance rates for individual MCMC walkers for *Eomes* in differentiating mouse e13.5 forebrain dorsal cells, Related to STAR Methods. The boxplots highlight the distribution of acceptance rates for each walker in each MCMC run. Walkers with the lowest acceptance rates (plotted in orange) are pruned and discarded from downstream analyses.

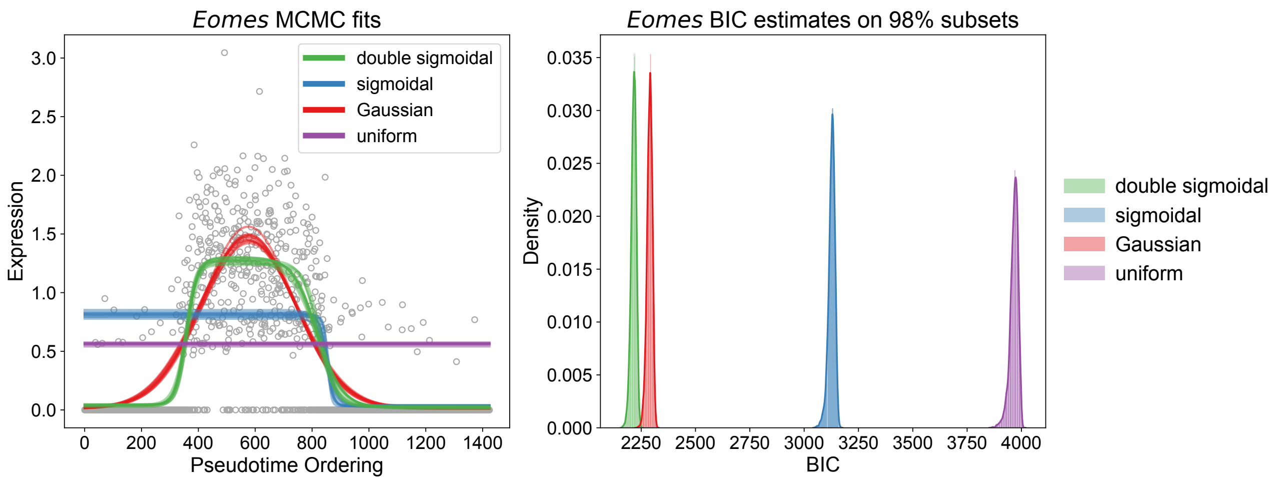

Figure S12: MCMC fits and BIC estimates for *Eomes* in differentiating mouse e13.5 forebrain dorsal cells, Related to STAR Methods. The left panel in the plot displays a random sampling of the parameters from 100 iterations from the MCMC traces across all samplers for the double sigmoidal, Gaussian, sigmoidal and uniform MCMC runs, as well as the expression levels of *Eomes* in cells ordered according to their relative pseudotemporal ordering. The right panel highlights the BIC estimates over 10,000 subsets of the data removing a random 2% of the data. Using this approach, the doube sigmoidal model is selected as the best-fitting model.

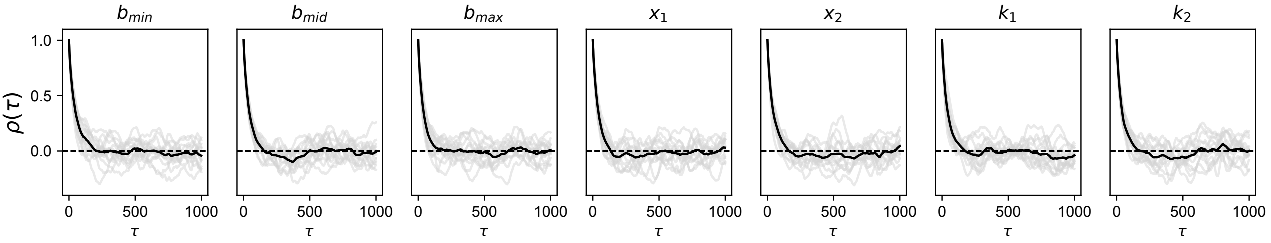

Figure S13: $\rho_f(\tau)$ **estimates in MCMC double sigmoidal run for *Eomes* in differentiating mouse e13.5 forebrain dorsal cells, Related to STAR Methods.** The autocorrelation function, $\rho_f(\tau)$, is displayed in the y-axis at time lags $\tau \in [0, 1000]$ for each parameter across all MCMC walkers after removing the first 5,000 iterations (burn-in), and removing the samplers in the bottom half of acceptance rates. Note that the autocorrelation function decays to 0 after a certain time delay, τ. Individual walkers are displayed in gray, with the average across all walkers in black.

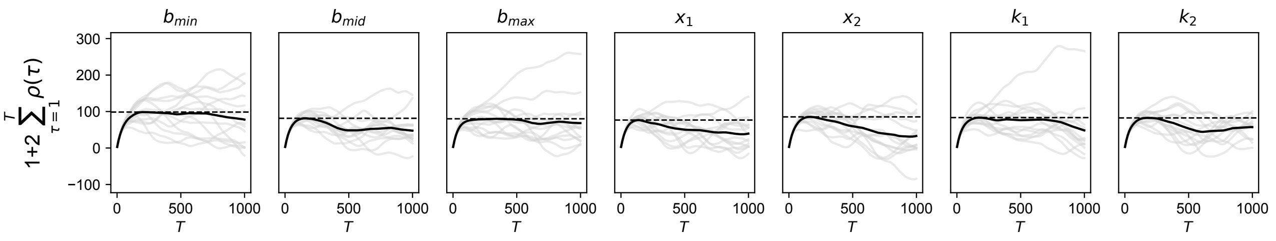

Figure S14: $\hat{\tau}_f$ estimates in MCMC double sigmoidal run for *Eomes* in differentiating mouse e13.5 forebrain dorsal cells, Related to STAR Methods. The integrated autocorrelation time, τ_f, is displayed in the y-axis estimated at time lags $T \in [0, 1000]$ for each parameter across all MCMC walkers after removing the first 5,000 iterations (burn-in), and removing the samplers in the bottom half of acceptance rates. Individual walkers are displayed in gray, with the average across all walkers in black. The final estimate, $\hat{\tau}_f$, as defined in, Equation 12, is highlighted with a dashed line. Note that the integrated autocorrelation time is quite similar across all parameters, and ranges from 76 to 99.

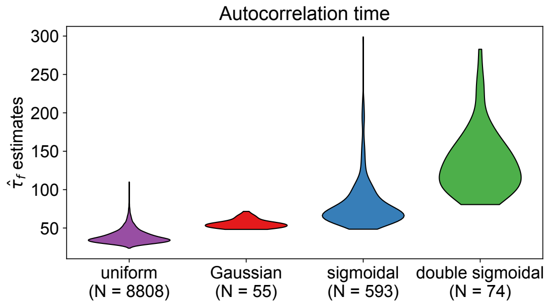

Figure S15: $\hat{\tau}_f$ estimates for all genes in differentiating mouse e13.5 forebrain dorsal cells, Related to STAR Methods. The violin plots display the distribution of autocorrelation time estimates for all genes, grouped by the best-fitting model. The number of genes in each category is displayed in the x-axis.

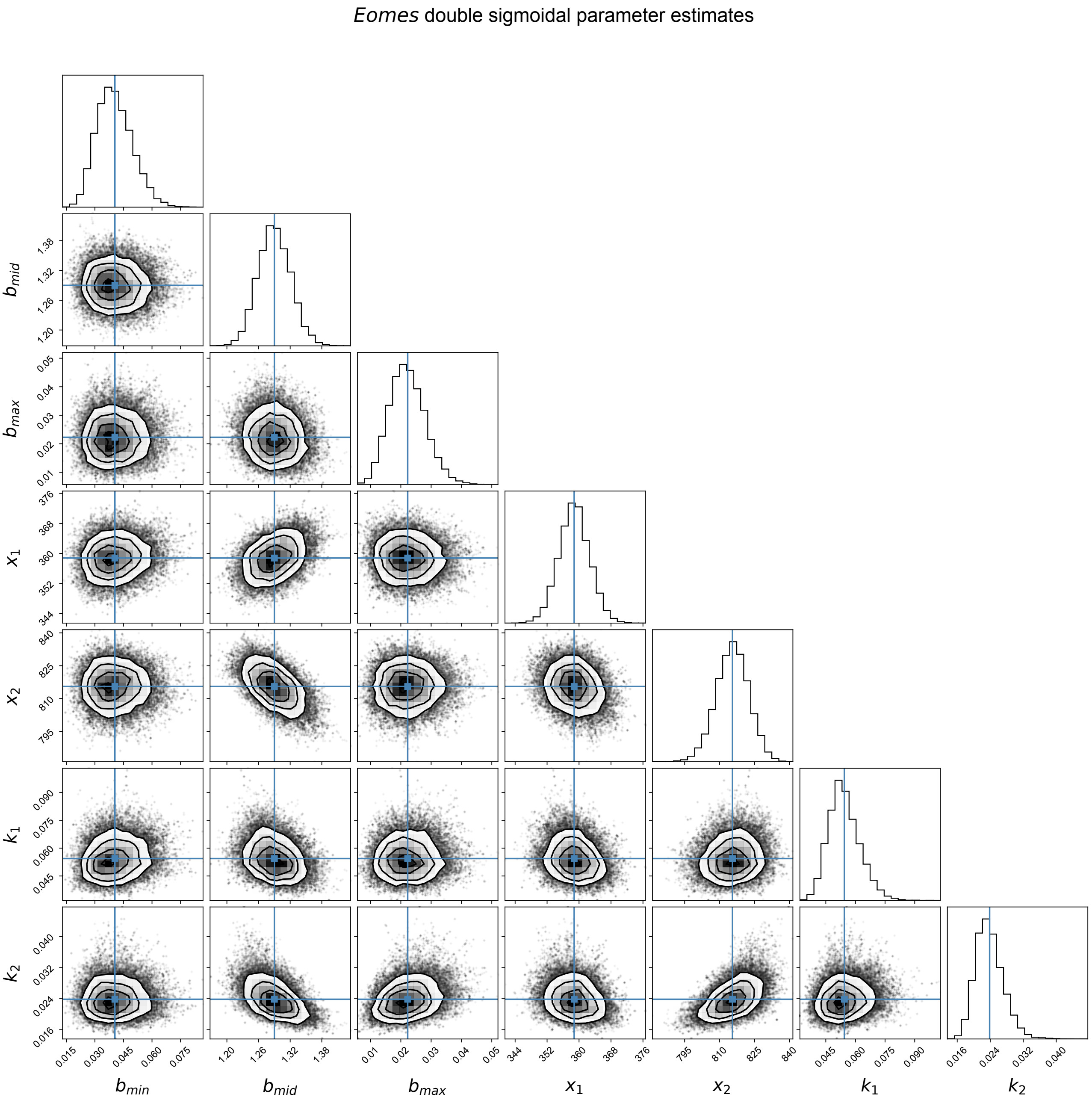

Figure S16: Corner plot of MCMC double sigmoidal run for *Eomes* in differentiating mouse e13.5 forebrain dorsal cells, Related to STAR Methods. The corner plot displays the two-dimensional projections (below the diagonal) and marginal distribution for individual parameters (on the diagonal) of the posterior distribution across all MCMC walkers after removing the first 5,000 iterations (burn-in), and removing the samplers in the bottom half of acceptance rates. The mean estimate for each parameter is highlighted in the horizontal and vertical lines.

Uniform (f_{unif})	Gaussian (f_{gauss})	Sigmoidal (f_{sig})	Double Sigmoidal (f_{dsig})
$b \sim \text{Unif}(0, \tilde{Y}_g)$	$b \sim \text{Unif}(0, \tilde{Y}_g)$	$b \sim \text{Unif}(0, \tilde{Y}_g)$	$b_{min} \sim \text{Unif}(0, \tilde{Y}_g)$
	$a \sim \text{Unif}(0, \tilde{Y}_g)$	$L \sim \text{Unif}(0, \tilde{Y}_g)$	$b_{mid} \sim \text{Unif}(0, \tilde{Y}_g)$
	$t_0 \sim \text{Unif}(1, N)$	$t_0 \sim \text{Unif}(1, N)$	$b_{max} \sim \text{Unif}(0, \tilde{Y}_g)$
	$\sigma \sim \mathcal{FN}(0, N/10)$	$k \sim \mathcal{FN}(0, 0.1)$	$t_1 \sim \text{Unif}(1, N)$
			$t_2 \sim \text{Unif}(1, N)$
			$k_1 \sim \mathcal{FN}(0, 0.1)$
			$k_2 \sim \mathcal{FN}(0, 0.1)$

Table S4: Priors on Function Parameters, Related to STAR Methods. $\tilde{Y}_g = \max(\{\tilde{y}_{gi}\}_{i=1,\dots,N})$ = maximum expression level for gene g in log-normalized space across all cells. Unif(a, b) refers to the uniform distribution on the open interval (a, b), and $\mathcal{FN}(\mu, \sigma^2)$ refers to a folded normal distribution with parameters μ and σ, as defined in Equation 8.
